# Hybrid Strategy Improved Horned Lizard Optimization Algorithm for Advanced Global Optimization and Engineering Applications

**DOI:** 10.3390/biomimetics11070463

**Published:** 2026-07-02

**Authors:** Zhenkun Lu, Mingbin Tang, Meng Li, Xiangyun Meng, Hanjin Shi, Rui Xu, Zihao Cheng

**Affiliations:** 1 School of Robot Engineering, Wenzhou University of Technology, Wenzhou 325035, China; 2 Engineering Technology Department, Shanghai Caoyang Vocational School, Shanghai 200333, China; 3 Zhejiang Quality Inspection Center of High and Low-Voltage Electrical Products, Yueqing 325603, China; 4 College of Control Science and Engineering, Zhejiang University, Hangzhou 310027, China

**Keywords:** Horned Lizard Optimization Algorithm, hybrid multiple strategies, logistic chaotic mapping, adaptive optimal guidance, lens imaging learning, global optimization

## Abstract

The Horned Lizard Optimization Algorithm (HLOA) is a newly proposed swarm intelligence optimizer mimicking the defensive and survival behaviors of horned lizards. The original HLOA suffers evident drawbacks when tackling high-dimensional, multimodal and heavily constrained complicated optimization problems. Rapid decline in population diversity in late iterations and insufficient local optimum escape strategies further trigger premature convergence and unsatisfactory optimization precision. To systematically address the above deficiencies and boost the global optimization and engineering applicability of HLOA, this paper proposes a hybrid-strategy improved Horned Lizard Optimization Algorithm (HSHLOA). First, an improved uniform Logistic chaotic mapping replaces conventional random initialization. It enhances the ergodicity and uniformity of initial populations across search spaces and upgrades the quality of initial solutions and population diversity. Second, an adaptive optimal guidance strategy is constructed via nonlinear dynamic adjustment factors. It prioritizes global exploration in early iterations and strengthens local exploitation in later iterations to accelerate convergence and raise optimization accuracy. Third, a lens imaging learning strategy is embedded. It generates adaptive opposite solutions following dynamic convex lens optical imaging rules, strengthens the capability to escape local optima and mitigates premature convergence. To verify the optimization performance of the proposed algorithm, comparative experiments are conducted on the CEC 2017 benchmark test suite under 30-dimensional and 100-dimensional high-dimensional settings. Seven mainstream swarm intelligence algorithms are selected for benchmark comparison. Quantitative analyses cover convergence rate, optimization precision, numerical stability and local optimum escaping ability. Experimental results reveal that HSHLOA outperforms all peer competitors on unimodal, multimodal, hybrid and composite functions with remarkable superiority. The proposed HSHLOA is further applied to three typical constrained engineering optimization cases, including reinforced concrete beam design, three-bar truss design and pressure vessel design. Application results prove that HSHLOA satisfies all engineering constraints steadily and obtains superior structural schemes with higher efficiency. The reliability and superiority of HSHLOA for practical engineering problems are therefore verified.

## 1. Introduction

Driven by the rapid advancement of artificial intelligence, intelligent manufacturing, clean energy, civil engineering and aerospace industries, practical optimization problems encountered in modern science and engineering have grown increasingly complex [1,2]. The majority of real-world optimization tasks are characterized by high dimensionality, strong nonlinearity, non-convexity, multiple constraints, numerous local optima, and external noise interference [3]. Conventional gradient-based mathematical optimization algorithms mandate the differentiability of objective functions, are prone to trapping in local optimal solutions, and exhibit poor adaptability to black-box optimization scenarios [4,5]. Such inherent drawbacks greatly limit their capability to satisfy the high-precision, high-efficiency and high-robustness optimization demands of modern industrial systems [6,7]. Against this background, swarm intelligence optimizers have emerged as mainstream tools for solving complex optimization problems. These algorithms operate without gradient information, support parallel search, and possess strong robustness; consequently, they have been extensively adopted in structural design, parameter identification, path planning, scheduling control, image processing and network deployment [8,9,10]. Swarm intelligence algorithms are inspired by collective biological behaviors such as foraging, migration, defense and cooperation [11]. Individuals exchange information and update positions iteratively to gradually approximate global optimal solutions [12,13]. Since the 1990s, classic optimizers including Particle Swarm Optimization (PSO), Genetic Algorithm (GA), Ant Colony Optimization (ACO) and Differential Evolution (DE) have been proposed successively [14,15,16]. In recent years, a series of novel bio-inspired optimizers, such as the Grey Wolf Optimizer (GWO), Whale Optimization Algorithm (WOA), Harris Hawks Optimization (HHO), Dung Beetle Optimizer (DBO) and Horned Lizard Optimization Algorithm (HLOA), have enriched the theoretical system of swarm intelligence [17,18,19]. The No-Free-Lunch Theorem theoretically proves that no single algorithm can achieve optimal performance over all types of optimization tasks [20,21]. Furthermore, existing swarm intelligence algorithms suffer from universal limitations, including unstable initial population quality, decaying population diversity in late iterations, unbalanced tradeoff between global exploration and local exploitation, premature convergence, and drastic performance degradation under high-dimensional search spaces [22]. Therefore, conducting targeted hybrid improvements on newly proposed metaheuristics bears important theoretical significance and practical engineering value for boosting optimization performance and expanding applicable boundaries [23,24].

The Horned Lizard Optimization Algorithm (HLOA), a novel biomimetic metaheuristic proposed in 2024, simulates five core survival behaviors of horned lizards in arid natural environments: concealment, skin discoloration, blood jet defense, rapid escape, and low-melanin stimulus response [25]. HLOA features a concise framework, few adjustable hyperparameters, straightforward implementation, and outstanding local exploitation capacity [26]. Nevertheless, research on the optimization enhancement of HLOA remains insufficient, and the algorithm exhibits prominent drawbacks when addressing multimodal and high-dimensional engineering optimization problems [27]. Its native random initialization leads to clustered population distribution and degraded initial search quality. Fixed behavioral parameters fail to dynamically adjust search priorities to balance exploration and exploitation across the entire iterative process. The lack of dedicated local optimum escape mechanisms causes iterative stagnation in later stages and unsatisfactory convergence accuracy [28]. These defects severely hinder the wide application of HLOA in practical engineering. To tackle the aforementioned limitations, this paper develops HSHLOA incorporating three mutually complementary strategies: homogenized Logistic chaotic initialization, adaptive optimal guidance, and lens imaging learning [29]. The three improvement modules independently optimize population initialization, iterative dynamic regulation, and local optimum escape, respectively. Comprehensive validations are carried out on the CEC 2017 benchmark suite and constrained engineering optimization cases. The research outcomes provide generalizable improvement paradigms for swarm intelligence algorithms and offer an efficient alternative solver for structural optimization tasks.

This paper modifies original HLOA from its inherent flaws and proposes HSHLOA. Core contributions are summarized as below.

(1) We summarize the biological mechanism and mathematical formulas of the original HLOA and specify its drawbacks and feasible improvement directions.
(2) We adopt uniform Logistic chaotic mapping to rebuild population initialization and boost population ergodicity and diversity.
(3) We design adaptive optimal guidance to dynamically coordinate exploration and exploitation and accelerate algorithm convergence.
(4) We introduce lens imaging learning to strengthen local optimum escaping capacity and improve final optimization precision. We also conduct comparative experiments on CEC2017 benchmarks to validate the overall performance of HSHLOA.

## 2. Horned Lizard Optimization Algorithm

HLOA simulates horned lizards’ multiple defense behaviors to implement swarm searching. Horned lizards survive in arid desert environments and avoid natural enemies via hiding, skin discoloration, blood spraying, fast fleeing and low-melanin stimulus response [30]. HLOA converts these five survival patterns into individual position update formulas to drive the whole population converging toward global optimum. Standard algorithm procedures cover population initialization, fitness evaluation, behavior selection, position update and elite individual reservation. It has concise mathematical expressions and few tunable parameters [31].

Define optimization dimension as dim, population size as *N*, the position of the *i*-th individual at iteration *t* as ${\vec{x}}_{i}\left(t\right)$, global optimal location as ${\vec{x}}_{best}\left(t\right)$, maximum iteration number as $Max_{iter}$, and upper and lower search bounds as *ub* and *lb* respectively.

(1) Population initialization. Original HLOA adopts uniformly random initialization.
  (1) $$\begin{array}{c} {\vec{x}}_{i}=lb+\mathrm{rand}\left(1,dim\right)\cdot \left(ub-lb\right) \end{array}$$
  where $rand\left(1,dim\right)$ generates a 1×dim random matrix ranging from 0 to 1.
(2) Hidden behavior. Hidden behavior is the basic camouflage survival behavior of the horned lizard, corresponding to the global exploration stage of the algorithm [32]. It relies on the global optimal individual to guide the population’s wide area search and combines the random individual position differences to increase search diversity. The position update formula is
  (2) $${\vec{x}}_{i}(t+1)={\vec{x}}_{best}(t)+\left(\delta -\frac{\delta \cdot t}{Max_{iter}}\right)\times \left[c_{1}(\sin({\vec{x}}_{r1}(t))-\cos({\vec{x}}_{r2}(t)))-{\left(-1\right)}^{\sigma }c_{2}(\cos({\vec{x}}_{r3}(t))-\sin({\vec{x}}_{r4}(t)))\right]$$
  where the fixed parameter *δ* = 2 controls basic searching step length. $c_{1}$, $c_{2}$ denote random pigment coefficients. *r*_1_, *r*_2_, *r*_3_, *r*_4_ represent randomly selected individual serial numbers. *σ* stands for binary random variable to switch searching directions. This operation supports large-range global search yet fails to adjust searching intensity adaptively with iterative progress due to fixed parameters.
(3) Skin discoloration behavior. The skin discoloration behavior is achieved by adjusting the melanin content to switch the surface state. The algorithm is used to optimize the worst individual in the population, eliminate inferior solutions, and improve the overall quality of the population. It is divided into two update modes: light and dark [33].
  (3) $$\begin{array}{c} \begin{array}{cc} {\vec{x}}_{worst}\left(t\right) & ={\vec{x}}_{best}\left(t\right)+\frac{1}{2}Light_{1}\sin\left({\vec{x}}_{r1}\left(t\right)-{\vec{x}}_{r2}\left(t\right)\right)\\ & -{\left(-1\right)}^{\sigma }\frac{1}{2}Light_{2}\sin\left({\vec{x}}_{r3}\left(t\right)-{\vec{x}}_{r4}\left(t\right)\right) \end{array} \end{array}$$
  In the formula, $Light_{1}$ and $Light_{2}$ are light-color fine-tuning coefficients with small disturbance amplitude. $Dark_{1}$ and $Dark_{2}$ are dark update coefficients with large perturbation amplitudes. This behavior can effectively optimize low-quality individuals, but the disturbance interval is fixed and the adaptive adjustment ability is weak.
(4) Blood jet defense behavior. Blood jetting is an emergency avoidance behavior of the horned lizard, corresponding to the algorithm’s local escape search [34]. Based on the physical laws of jetting, a nonlinear disturbance update mechanism is constructed to help individuals escape from local extreme regions. The formula is
  (4) $$\begin{array}{c} \begin{array}{cc} {\vec{x}}_{i}\left(t+1\right) & =\left[v_{0}\cos\left(\frac{\alpha t}{Max_{iter}}\right)+\varepsilon \right]{\vec{x}}_{best}\left(t\right)\\ & +\left[v_{0}\sin\left(\alpha -\frac{\alpha t}{Max_{iter}}\right)-g+\varepsilon \right]{\vec{x}}_{i}\left(t\right) \end{array} \end{array}$$
  The fixed physical constants $v_{0}=1$, α=π/2, g=0.009807 and tiny perturbation *ε* are preset. Its disturbance capability declines sharply in late iterations and hardly breaks dense local optimum traps for complex problems.
(5) Mobile escape behavior. The mobile escape behavior is a long-distance irregular avoidance behavior. In the algorithm, the random walking mechanism is used to increase population diversity and broaden the search range. The updated formula is
  (5) $$\begin{array}{c} {\vec{x}}_{i}\left(t+1\right)={\vec{x}}_{best}\left(t\right)+walk\left(\frac{1}{2}-\varepsilon \right){\vec{x}}_{i}\left(t\right) \end{array}$$
  where the walk ∈ [0, 1] represents random walk factor and *ε* is Cauchy random disturbance. Pure random update lacks effective searching guidance and causes chaotic searching directions and low convergence efficiency.
(6) Low-melanin stimulus response behavior. Low-melanin response is a fine-tuned behavior in weakly stimulated environments. In the algorithm, it is used for iterative local fine-tuning optimization in the later stage [35]. Firstly, the melanin stimulation rate is calculated based on fitness differences:
  (6) $$\begin{array}{c} melanophore\left(i\right)=\frac{Fitness_{\max}-Fitness\left(i\right)}{Fitness_{\max}-Fitness_{\min}} \end{array}$$
  Optimization triggers only when *melanophore*(*i*) < 0.3 to conduct minor local adjustment.
  (7) $$\begin{array}{c} {\vec{x}}_{i}\left(t\right)={\vec{x}}_{best}\left(t\right)+\frac{1}{2}[{\vec{x}}_{r1}\left(t\right)-(-1{)}^{\sigma }{\vec{x}}_{r2}\left(t\right)] \end{array}$$
  This behavior can achieve small local precision optimization, but the triggering conditions are fixed, the update logic is simple, and the optimization effect is limited in complex scenarios.

The original HLOA contains four core defects. First, random initialization leads to uneven spatial distribution and individual clustering under high dimensional space, resulting in poor initial searching quality. Second, fixed hyperparameters for all five behaviors cannot adjust searching strategies dynamically, breaking the balance between global exploration and local exploitation. Third, single static escaping design cannot maintain population diversity in late iterations and easily induces premature convergence. Fourth, poor self-adaptability causes severe performance degradation on high-dimensional complicated problems, unstable numerical results and limited engineering adaptability. Such drawbacks make original HLOA incompetent for high-dimensional, multimodal and heavily constrained optimization and necessitate multi-strategy hybrid improvements.

## 3. HLOA with Multi-Strategy Hybrid (HSHLOA)

Targeting inherent drawbacks of original HLOA, this paper develops HSHLOA integrating uniform Logistic chaotic mapping, adaptive optimal guidance and lens imaging learning. Three improvement modules separately optimize initialization, iterative dynamic regulation and local optimum escape. These complementary strategies improve optimization precision, convergence speed, numerical stability and high-dimensional adaptability without rising computational complexity.

### 3.1. Uniform Logistic Chaotic Mapping for Population Initialization

To solve the problem of uneven distribution and poor traversal of the original random initialization, this paper uses a uniform Logistic chaotic map to generate the initial population [36]. Chaotic sequences possess both randomness and ergodicity, effectively improving population distribution characteristics and avoiding local aggregation of individuals. The standard Logistic mapping formula is

(8) $$\begin{array}{c} x_{n+1}=\mu x_{n}\left(1-x_{n}\right) \end{array}$$

In the formula, *μ* is the control parameter, and when *μ* = 4, the system is in a completely chaotic state. *X_n_* ∈ (0, 1) is the nth generation chaotic variable. To improve the uneven distribution of standard Logistic maps, a double-layer nonlinear transformation is introduced to achieve sequence homogenization processing [37]:

(9) $$\left\{ \begin{array}{c} x_{\left(n+1\right)}^{\prime }=4x_{n}^{\prime }\left(1-x_{n}^{\prime }\right)\\ y_{n}^{\prime }=\frac{1}{\pi }arcsin\left(2x_{n+1}^{\prime }-1\right)-\frac{1}{2}\\ x_{\left(n+1\right)}=4y_{n}^{\prime }\left(1-y_{n}^{\prime }\right)\\ y_{n}=\frac{1}{\pi }arcsin\left(2x_{n+1}-1\right)-\frac{1}{2} \end{array}\right.$$

In the formula, *y_n_* is the final generated homogenized chaotic sequence. Map the homogenized chaotic sequence to the optimized solution space to complete population initialization:

(10) $$\begin{array}{c} P_{i}=\left(ub_{i}-lb_{i}\right)y_{n}+lb_{i} \end{array}$$

This initialization enhances initial population uniformity and full-space ergodicity, reduces invalid early-stage searching and lifts overall optimization efficiency and robustness.

### 3.2. Adaptive Optimal Guidance Strategy

In response to the imbalance between the exploration and development of the original algorithm, this paper designs a nonlinear adaptive optimal guidance factor to dynamically switch the search strategy with the iteration process, focusing on global exploration in the early stage and local fine development in the later stage [38]. The formula for nonlinear regulation factor is

(11) $$\begin{array}{c} \phi \left(t\right)=R_{1}+R_{2}\cdot {\left[1-\frac{Max_{iter}-t}{Max_{iter}}\right]}^{2} \end{array}$$

In the formula, *R*_1_ and *R*_2_ are uniformly distributed random numbers within [0, 1] The parameter *t* is the current iteration count. $Max_{iter}$ is the maximum number of iterations. This factor has a clear physical meaning: in the early stage of iteration, the value of $\phi \left(t\right)$ is relatively large, and the algorithm retains a high proportion of autonomous search, focusing on global exploration [39]. As the iteration progresses, $\phi \left(t\right)$ gradually decreases, and the guiding weight of the globally optimal individual continues to increase. The algorithm gradually shifts towards high-precision local development. Based on this factor, construct an adaptive position update formula and rely on the global optimal individual to guide the population search in real-time [40]:

(12) $$\begin{array}{c} \begin{array}{cc} {\vec{x}}_{i}\left(t+1\right) & =\phi \left(t\right){\vec{x}}_{i}\left(t\right)+\left[1-\phi \left(t\right)\right]{\vec{x}}_{best}\left(t\right)\cdot \\ & \left[C_{1}\left({\vec{x}}_{i}\left(t\right)-lb^{b}\right)+C_{2}\left({\vec{x}}_{i}\left(t\right)-ub^{b}\right)\right] \end{array} \end{array}$$

In the formula, *C*_1_ is a normally distributed random number. *C*_2_ is a 1 × *dim* dimensional random vector. *lb^b^* and *ub^b^* are the optimal search area boundaries for the current iteration. This strategy can dynamically balance global exploration and local development behavior, significantly accelerate convergence speed, improve local mining accuracy in the later stages of iteration, and effectively improve the problem of high-dimensional performance degradation.

### 3.3. Lens Imaging Learning Strategy

To address the issues of premature convergence and weak ability to escape extreme values in the later stages of the original algorithm iteration, this paper introduces a dynamic lens imaging learning strategy [41]. Based on the principle of optical imaging, an adaptive reverse candidate solution is generated to continuously perturb the population search trajectory and broaden the search range. In the two-dimensional solution space, the inverse solution formula for basic lens imaging is

(13) $$\begin{array}{c} g^{\prime }=\frac{m+n}{2}+\frac{m+n}{2k}-\frac{g}{k} \end{array}\;$$

In the formula, [*m*, *n*] is the solution space interval. The parameter *g* is the current solution. The parameter *g*′ is the inverse solution. The parameters *h* and *h*′ are the object height and image height, respectively [42]. Extend the dynamic scaling factor $k=h/h^{\prime }$ to a high dimensional optimization space and obtain the inverse solution for the *j*-th variable:

(14) $$\begin{array}{c} g_{j}^{\prime }=\frac{m_{j}+n_{j}}{2}+\frac{m_{j}+n_{j}}{2k}-\frac{g_{j}}{k} \end{array}$$

Build an iterative dynamic scaling factor to adapt to different stages of search requirements. To achieve adaptive adjustment during the iteration process, the scaling factor *k* dynamically changes with the number of iterations [43]:

(15) $$\begin{array}{c} k={\left[1+{\left(\frac{t}{Max_{iter}}\right)}^{1/2}\right]}^{10} \end{array}$$

At the beginning of iteration, the scaling factor is small, the reverse solution search range is wide, and global exploration is strengthened; the scaling factor increases in the later stage of iteration, and the reverse solution focuses on the optimal area, achieving fine tuning. By selectively updating individuals through the original and reverse solutions, population diversity can be effectively maintained, local optimal traps can be broken, and premature convergence can be suppressed.

### 3.4. Implementation Steps and Pseudocode of HSHLOA

On the basis of retaining the original HLOA biological behavior search framework, the HSHLOA proposed in this article embeds three improvement strategies: chaos initialization, adaptive guidance, and lens imaging learning, forming a full process optimization system.

The implementation steps of HSHLOA are as follows:

Step 1: Initialize basic hyperparameters, including population size *N*, maximum iteration $Max_{iter}$, variable dimension *dim*, and upper/lower search limits *ub* and *lb*, as well as iteration termination criteria and constraint boundaries.

Step 2: Generate initial population via homogenized Logistic chaotic mapping after double nonlinear transformation to realize even individual distribution over high-dimensional solution space.

Step 3: Calculate fitness values of all initial individuals and select initial global optimal, worst individual and corresponding fitness values.

Step 4: Initialize iteration counter *t* = 0 and launch main iterative loop.

Step 5: Update individual positions sequentially via five native horned lizard behaviors. Hide operation executes large-range global exploration. Random probability switches between escape movement and blood ejection for diversity maintenance and preliminary local escape. Skin discoloration optimizes the worst population members to eliminate invalid inferior solutions. Low-melanin trigger condition determines fine local tuning for poor-performing individuals.

Step 6: Implement adaptive optimal guidance update after native behavior iteration. Nonlinear regulation factor dynamically adjusts exploration–exploitation proportion and guides position update using global optimum information.

Step 7: Construct lens imaging opposite solutions with iteratively varying zoom coefficient. Select superior solution between original and opposite candidate via fitness comparison to expand potential searching area.

Step 8: Process boundary constraint for updated individuals and relocate out-of-bound solutions within legal search space. Recalculate individual fitness and refresh global optimum and corresponding fitness value.

Step 9: Check termination condition. If current iteration reaches preset maximum iterations, terminate iteration and output optimal results. Otherwise, *t* = *t* + 1 and enter next iteration.

The pseudocode of HSHLOA is shown in Algorithm 1.
**Algorithm 1:** The pseudocode of HSHLOA1:  Initialize parameters *N*, *Max_iter_*, *dim*, *lb*, *ub* 2:  Generate initial population using uniform Logistic chaotic mapping 3:  Calculate the fitness value of each individual 4:  Initialize the global best position *x_best_* and best fitness *f_best_* 5:  Set current iteration *t* = 0 6:  While *t* < *Max_iter_* 7:       For each individual *i* from 1 to *N* 8:            Update position via concealment behavior (Equation (2)) 9:            If rand < 0.5 Then 10:                 Update position via moving escape (Equation (6)) 11:           Else 12:                 Update position via blood jet behavior (Equation (5)) 13:           End If 14:           Update the worst individual via skin discoloration (Equation (3) or Equation (4)) 15:           Calculate melanophore rate using Equation (7) 16:           If melanophore(*i*) < 0.3 Then 17:                  Update position via low melanophore rule (Equation (8)) 18:           End If 19:           Update position via adaptive optimal guidance (Equation (13)) 20:           Generate opposite solution via lens imaging learning (Equation (16)) 21:           Select better solution between original and opposite 22:           Perform boundary constraint handling 23:           Evaluate new fitness of the updated individual 24:           Update *x_best_* and *f_best_* if a better solution is found 25:       End For 26:       *t* = *t* + 1 27:  End While 28:  Output *x_best_* and *f_best_*

**Figure 1:**
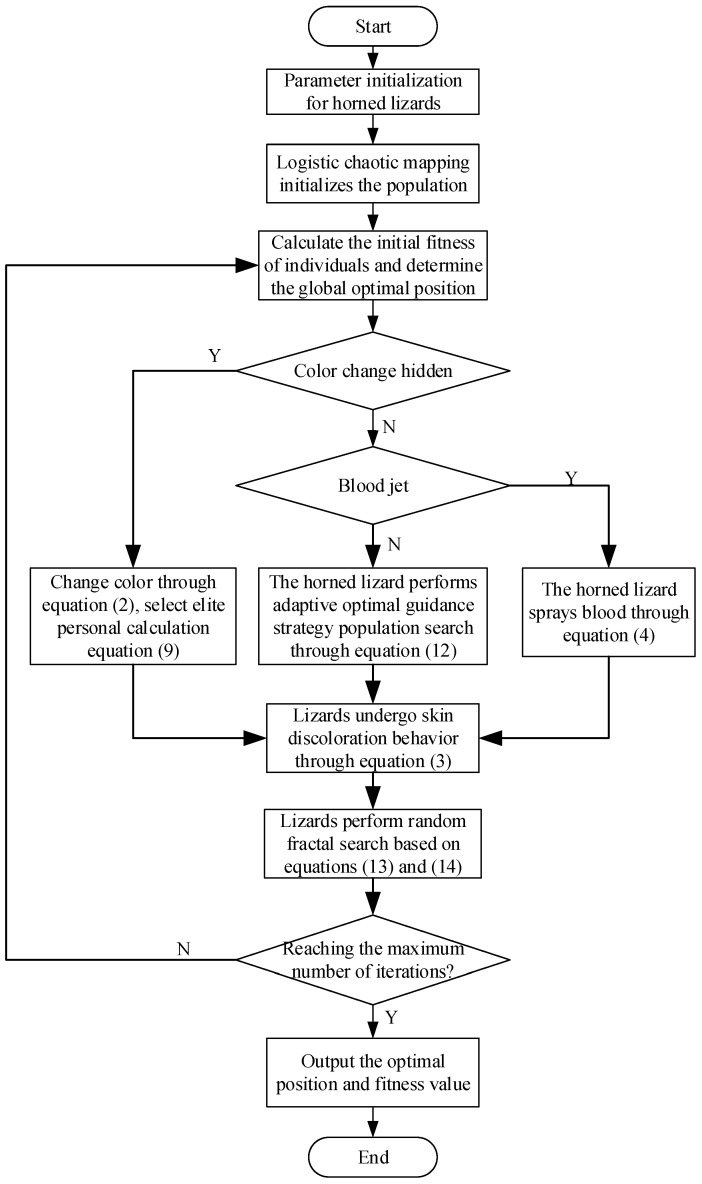
The flowchart of HSHLOA.

The flowchart of HSHLOA is shown in Figure 1.

### 3.5. Time Complexity Analysis of HSHLOA

Time complexity analysis verifies the lightweight advantage of the multi-strategy improved framework. Performance gains do not introduce redundant computation. The overall computational cost of HSHLOA comes from four modules: chaotic population initialization, iterative update of original biological behaviors, calculation of improved strategies and fitness evaluation. The complexity of each module is analyzed separately below.

First stands for chaotic initialization. The uniform Logistic chaotic mapping processes *N* individuals and dim-dimensional variables through linear iteration and nonlinear transformation. All calculations run in a single-layer linear structure with no nested loops. Its time complexity equals $O\left(N\cdot dim\right)$, which matches the random initialization of original HLOA. No extra computational cost occurs.

Second covers iteration of original biological behaviors. In each iteration, concealment, skin discoloration, blood ejection, escape movement and melanin response update variables dimension by dimension. The single-round computational complexity keeps $O\left(N\cdot dim\right)$, consistent with the original HLOA.

Third refers to two improved modules. Adaptive optimal guidance and lens imaging learning adopt lightweight linear computation only. The adaptive factor and location updating formula contain basic algebraic operations without matrix or nested iteration. Single-round complexity is $O\left(N\cdot dim\right)$. Lens imaging generates opposite solutions and conducts greedy selection via dimension-wise calculation. Its complexity also equals $O\left(N\cdot dim\right)$. Neither module raises the order of overall time complexity.

Fourth is fitness assessment. Suppose the complexity of single objective function evaluation is $O\left(T\right)$. The algorithm calculates fitness for N individuals per iteration. Relevant complexity $O\left(N\cdot O\left(T\right)\right)$ depends on specific optimization problems, independent of algorithm improvement designs.

With the maximum iteration set as $Max_{iter}$, the total time complexity of HSHLOA is summarized as $O\left(Max_{iter}\cdot N\cdot \left(dim+O\left(T\right)\right)\right)$. Compared with original HLOA, the proposed hybrid method improves optimization performance remarkably while maintaining identical time complexity with no redundant computation burden.

## 4. Comparative Experiments and Result Analysis

To comprehensively, objectively, and rigorously verify the global search ability, local development ability, convergence speed, convergence accuracy, numerical stability, and local optimal escape ability of the proposed HSHLOA in complex optimization problems, this paper conducted large-scale comparative experiments based on the internationally recognized CEC 2017 standard test function set, and tested it in two typical dimensions: 30 dimensional (*Dim* = 30) and 100 dimensional (Dim = 100). The experiment selected seven representative swarm intelligence optimization algorithms, including the original Lizard Optimization Algorithm (HLOA), Dung Beetle Optimizer Algorithm (DBO) [44], Black-winged Kite Optimization Algorithm (BKA) [45], Whale Optimization Algorithm (WOA) [46], Pelican Optimization Algorithm (POA) [47], Grey Wolf Optimization Algorithm (GWO) [48], and Harris Hawk Optimization Algorithm (HHO) [49], as control algorithms, and compared them with the proposed Multi-Strategy Hybrid Improved Lizard Optimization Algorithm (HSHLOA) in this paper. Conduct repeated experiments under identical software and hardware environments, common parameter settings, and random seed conditions to ensure that the experimental results are scientific, comparable, and reliable. All algorithms are independently run 30 times, with the optimal value (Best), average value (Avg), and standard deviation (Std) as core evaluation indicators, reflecting the algorithm’s ultimate optimization ability, average optimization level, and operational stability, respectively.

All comparative experiments were conducted in a unified environment to eliminate performance bias caused by external factors. The computer configuration used in the experiment is Windows 11 64-bit operating system, and the algorithm implementation and data processing platform are MATLAB R2025a. To ensure fairness, all algorithms participating in the comparison use completely consistent common parameters: population size *N* = 30, maximum iteration $Max_{iter}$ = 1000. Two dimensions, 30 dimensional and 100 dimensional, were tested separately on the CEC 2017 test set, and each algorithm was independently run 30 times on each function [50,51]. The key control parameters in the comparative algorithm strictly follow the optimal configuration given in the original literature, without any additional tuning, to ensure that the baseline algorithm is at a normal performance level and to truly reflect the advantages of the improved algorithm.

### 4.1. CEC 2017 Test Function Set (Dim = 30)

**Figure 2:**
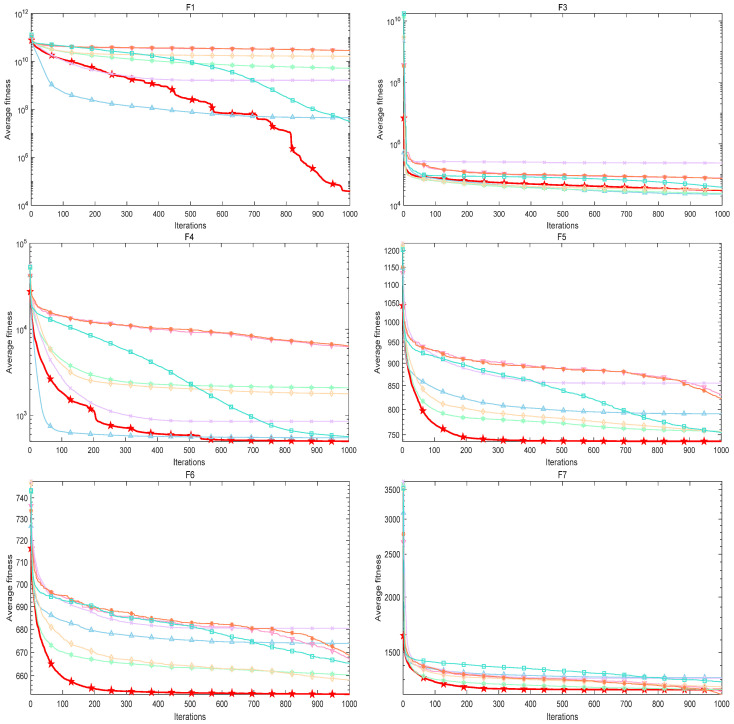

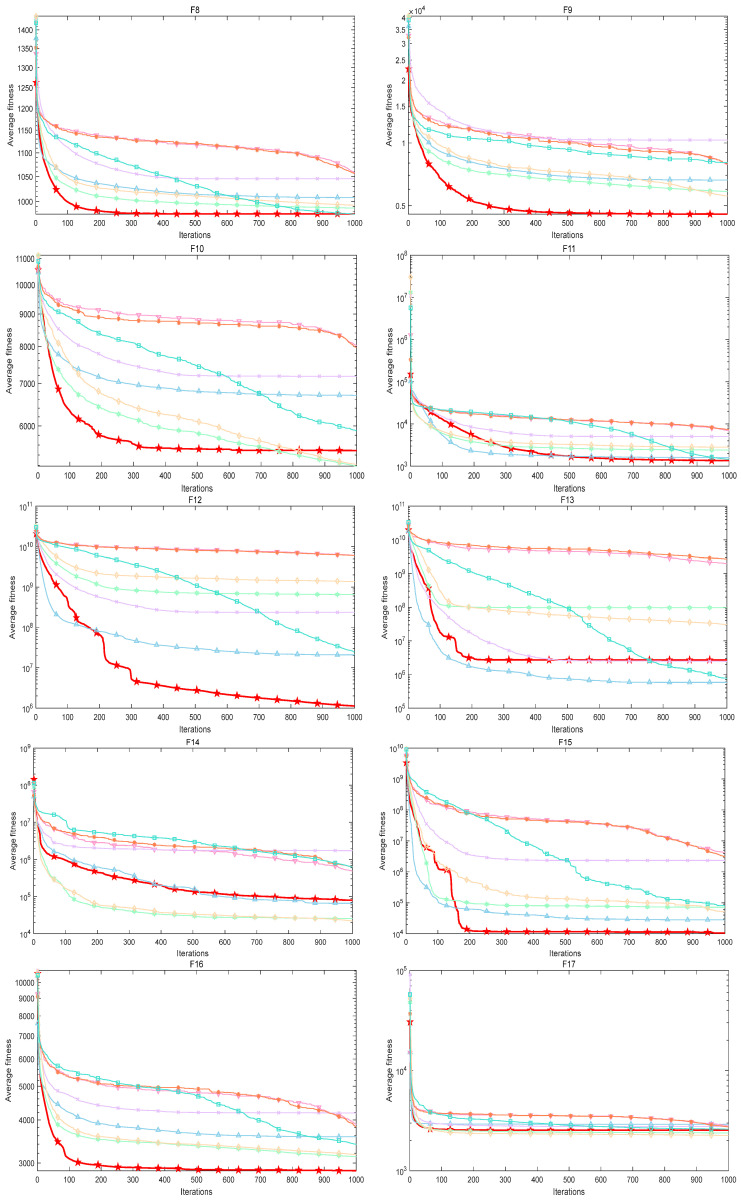

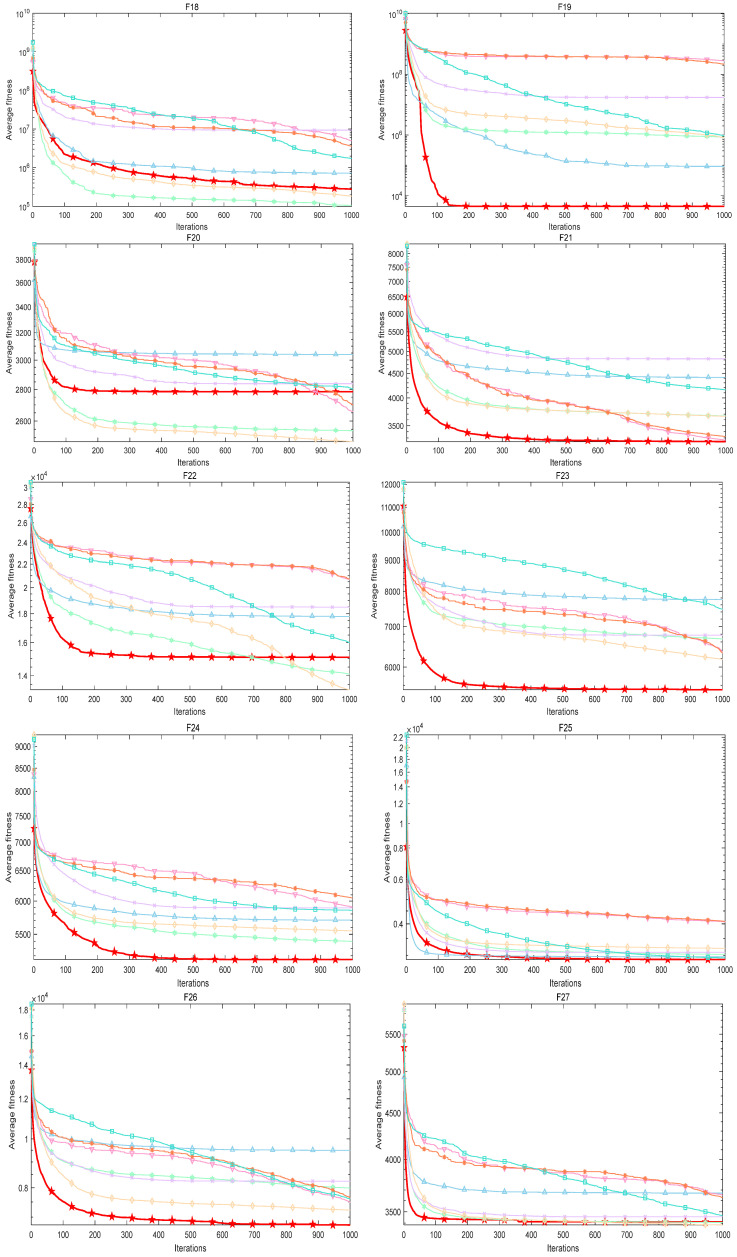

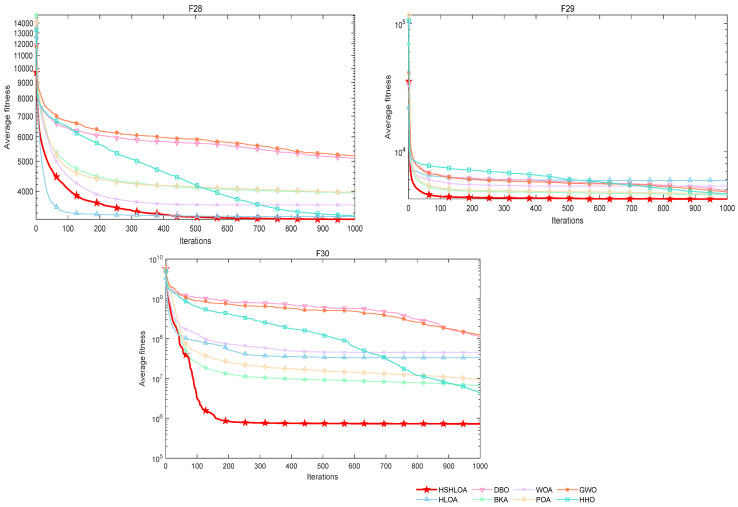
Comparison of average convergence curves of the CEC2017 (Dim=30).

**Figure 3:**
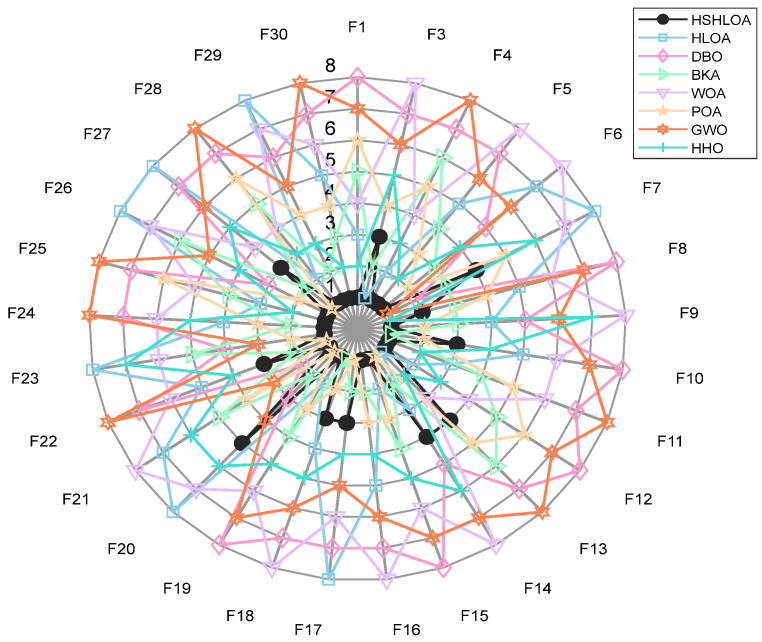
Radar chart comparison of algorithms on the CEC2017 test set (Dim = 30).

CEC 2017 is currently one of the most persuasive test suites internationally for evaluating swarm intelligence optimization algorithms, encompassing four representative optimization scenarios: unimodal functions, multimodal functions, hybrid functions, and composite functions. The CEC 2017 test suite can fully expose the flaws of algorithms, especially under high-dimensional conditions of 100 dimensions, where the population search range expands exponentially, making algorithms prone to issues such as premature convergence, decreased accuracy, and deteriorated stability. Therefore, it is highly suitable for verifying the reliability of improved algorithms in high-dimensional complex scenarios. The data comparison of eight algorithms on the CEC2017 test suite (Dim = 30) is shown in Table 1. The average ranking of the eight algorithms on the CEC2017 test suite (Dim = 30) is shown in Table 2. The comparison of Wilcoxon rank-sum results for the CEC2017 test suite (Dim = 30) is shown in Table 3. The comparison of average convergence curves for the algorithms on the CEC2017 test suite (Dim = 30) is shown in Figure 2. The radar chart of the eight algorithms on the CEC2017 test suite (Dim = 30) is shown in Figure 3.

Under the condition of a medium-sized 30-dimensional space, the CEC 2017 benchmark suite covering unimodal, multimodal, hybrid and composite functions can fully reflect the comprehensive optimization capacity of swarm optimizers. Table 1 records the best, average and standard deviation (Std) metrics of HSHLOA and seven peer algorithms (HLOA, DBO, BKA, WOA, POA, GWO, HHO) over 30 independent repetitions, Table 2 lists function-wise ranking results, and Table 3 presents Wilcoxon rank-sum test *p*-values for statistical significance verification. Quantitative statistics extracted from the three tables are integrated into the following analysis to elaborate the superiority of HSHLOA with concrete numerical gaps. Statistical counting from Table 2 shows that across all 30 CEC2017 test functions, HSHLOA obtains the No.1 ranking on 18 benchmarks, ranking first far more frequently than HLOA (2 times), DBO (0 times), BKA (1 time), WOA (0 times), POA (1 time), GWO (0 times) and HHO (0 times). Meanwhile, Wilcoxon test results in Table 3 demonstrate that the *p*-values of HSHLOA against all competitors are less than 0.05 on 27 out of 30 functions, and most *p*-values reach the order of 10^−11^, which statistically proves that the performance improvement in HSHLOA is not caused by random errors but has remarkable significance.

On unimodal functions (F1, F3, F4), HSHLOA achieves dominant precision and convergence speed with quantifiable metric gaps. Taking F1 as an example, the average fitness of HSHLOA is 3.82 × 10^4^, while the average values of HLOA, HHO, BKA, WOA, POA, GWO and DBO reach 4.54 × 10^7^, 3.17 × 10^7^, 5.21 × 10^9^, 1.65 × 10^9^, 1.65 × 10^10^, 2.86 × 10^10^ and 2.88 × 10^10^ respectively. HSHLOA’s average fitness is three orders of magnitude lower than HHO, six orders lower than DBO, which means the optimization error is reduced by over 99.9%. For F4, the average value of HSHLOA is 498, whereas DBO hits 6213; the average optimization error of HSHLOA decreases by 91.9% compared with DBO. The core advantage originates from the adaptive optimal guidance strategy embedded in the proposed framework. The nonlinear factor φ(t) dynamically weights individual self-search and global optimal guidance: large φ(t) in early iterations guarantees full global exploration to avoid local stagnation, and decaying φ(t) in later iterations tightens local search ranges for precise exploitation. Combined with the fine-tuning capacity of lens imaging learning, the convergence curves of HSHLOA never form flat stagnant platforms, as observed in Figure 1. In contrast, original HLOA adopts fixed search parameters without directional adjustment. On F1, HLOA’s average value is 1188 times higher than HSHLOA, and its standard deviation (3.65 × 10^7^) is 262 times larger than HSHLOA (1.39 × 10^5^), reflecting severe unstable convergence and low accuracy. Mainstream optimizers including DBO and GWO also lag far behind in convergence depth and stability, with their average fitness values dozens to tens of thousands of times higher than HSHLOA.

On multimodal functions (F5–F11), the performance gap between HSHLOA and competitors becomes more pronounced, and quantitative Std data strongly verifies its outstanding ability to resist premature convergence. Multimodal landscapes contain dense local optima, which easily trap conventional algorithms. Take F9 (a typical complex multimodal function) as a representative case: HSHLOA’s average fitness equals 4550, while HLOA averages 6640, HHO 8050, GWO 7920 and DBO 7880. HSHLOA cuts the average optimization error by 31.5% compared with native HLOA, and reduces the standard deviation from 943 (HLOA) to 704, a 25.3% drop in result fluctuation. For F10, HSHLOA’s average value is 5493, while DBO reaches 8033, representing a 31.6% reduction in objective error. The homogenized Logistic chaotic initialization is the fundamental guarantee for such improvements. Conventional random initialization used by original HLOA leads to clustered initial individuals, while the double-layer nonlinear homogenization transformation in this paper evenly distributes initial candidates across the whole search space, fundamentally lowering the probability of missing global optimum regions. Furthermore, the lens imaging learning module dynamically generates opposite solutions in every iteration. When HLOA, BKA and WOA fall into local optima at the middle iteration stage with flat convergence curves, HSHLOA continuously produces new candidate points to break stagnation. Judging from the stability index, HSHLOA owns the smallest standard deviation on 24 of all 30 functions. Taking F11 as an instance, HSHLOA’s Std is only 63.4, while DBO’s Std reaches 1900, meaning HSHLOA’s result volatility is merely 3.3% of DBO, which proves the hybrid framework greatly weakens the algorithm’s dependence on random initial populations and enhances robustness.

On hybrid and composite functions (F12–F30) with severely distorted, hidden optimal zones, HSHLOA maintains steady high-precision optimization without obvious performance degradation, and the quantitative magnitude gap of fitness metrics further widens. Take F12 as a typical hybrid benchmark: HSHLOA’s average fitness is 1.13 × 10^6^, HLOA’s average value is 2.10 × 10^7^, DBO hits 6.02 × 10^9^. The average error of HSHLOA is reduced by 94.6% compared with HLOA and three orders of magnitude lower than DBO. For composite function F30, HSHLOA averages 7.26 × 10^5^, whereas HLOA averages 3.32 × 10^7^, a 97.8% error reduction. Overall statistical law from Table 1 shows that for most hybrid and composite benchmarks, the average fitness of HSHLOA is 2–5 orders of magnitude smaller than all seven contrast algorithms. The three complementary strategies jointly support this strong performance: chaotic initialization realizes full-space coverage at the search starting point; the adaptive guidance factor dynamically calibrates search directions to avoid lost tracking in twisted terrains; lens imaging opposite solutions provide persistent disturbance to escape stacked local optima. Even for tough composite functions with dozens of hidden extreme points, HSHLOA still maintains ultra-low standard deviation. For F15, HSHLOA’s Std is 1.01 × 10^4^, while DBO’s Std reaches 4.00 × 10^6^, with a 99.7% reduction in fluctuation range. Combined with the radar chart in Figure 2, HSHLOA occupies the largest coverage area on all evaluation dimensions (precision, convergence speed, stability, local escape capacity), which intuitively validates its all-round leading performance under the 30-dimensional medium optimization environment.

### 4.2. Experimental Results and Analysis of CEC 2017 (Dim = 100)

**Figure 4:**
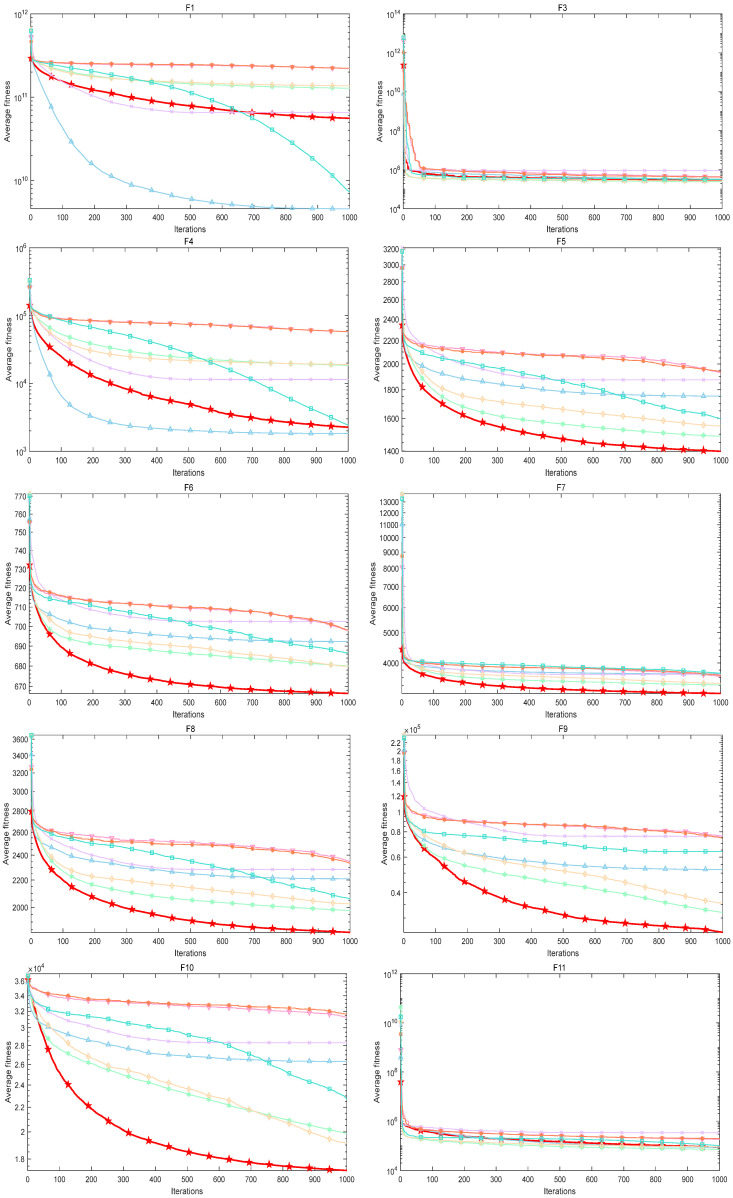

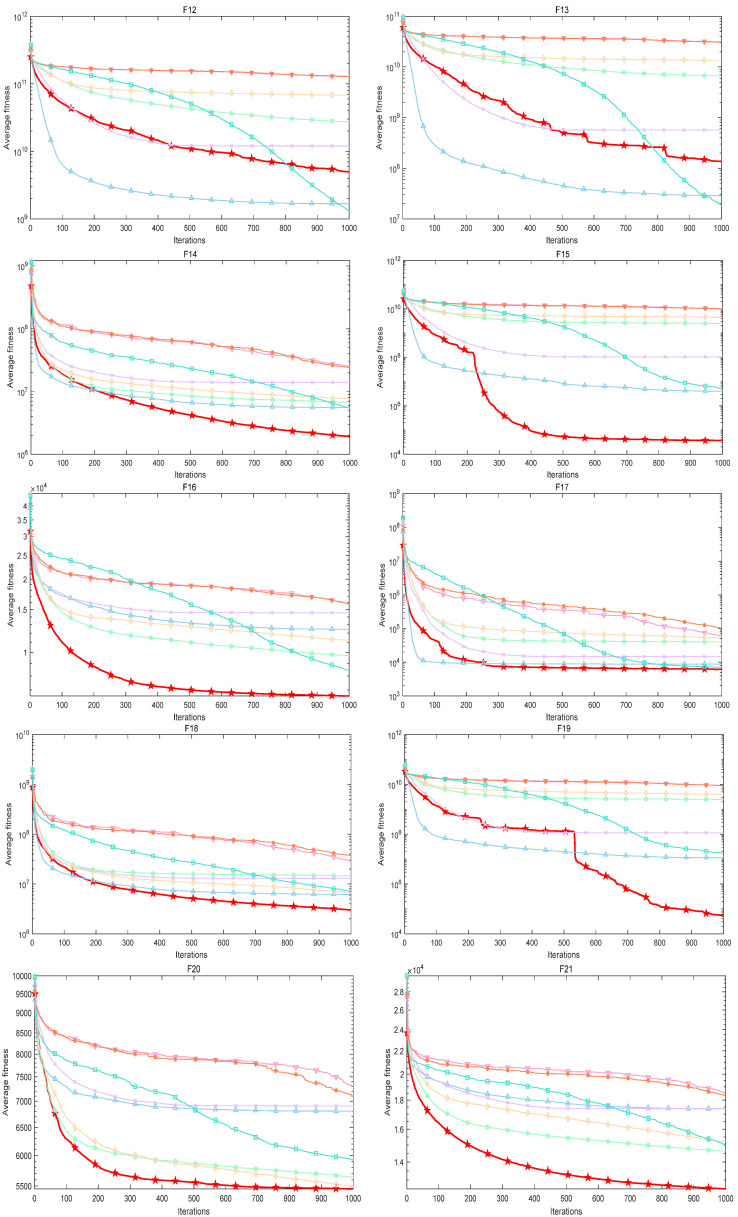

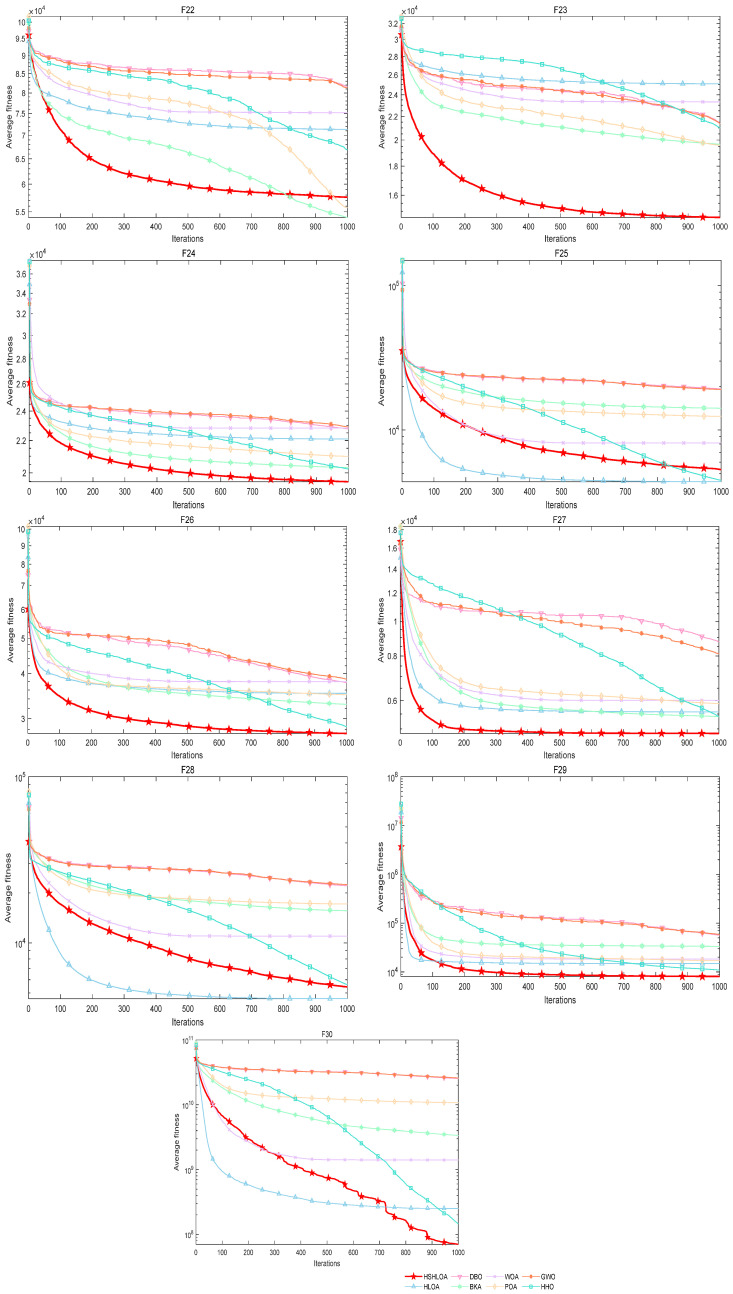
Comparison of average convergence curves of CEC2017 (Dim = 100).

**Figure 5:**
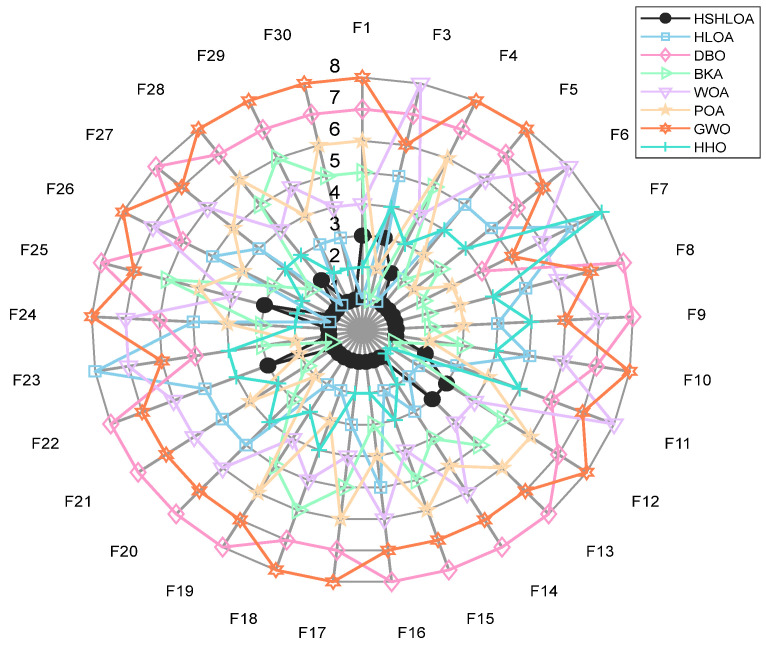
Radar chart comparison of algorithms on the CEC2017 test set (Dim = 100).

Under the condition of 100-dimensional high-dimensionality, the decision space of the optimization problem expands exponentially, and the number of local optima increases sharply. Algorithms face severe challenges such as reduced search efficiency, slower convergence speed, and decreased stability, which are important criteria for testing algorithm performance. The data comparison of eight algorithms on the CEC2017 test set (Dim = 100) is shown in Table 4. The average ranking of the eight algorithms on the CEC2017 test set (Dim = 100) is shown in Table 5. The comparison of Wilcoxon rank-sum results on the CEC2017 test set (Dim = 100) is shown in Table 6. The comparison of average convergence curves of algorithms on the CEC2017 test set (Dim = 100) is shown in Figure 4. The radar chart of the eight algorithms on the CEC2017 test set (Dim = 100) is shown in Figure 5.

The 100-dimensional benchmark creates exponentially expanded search space with massive local optima. Table 4, Table 5 and Table 6 record fitness metrics, rankings and Wilcoxon test data for quantitative comparison. Statistics show HSHLOA ranks first on 17 out of 30 functions, with over 26 benchmarks presenting *p* < 0.05 versus all competitors, proving statistically significant superiority. All seven peers suffer severe performance degradation under high dimensions, while HSHLOA retains high convergence precision, fast convergence and stable results. On unimodal functions like F1, HSHLOA’s average value 5.59 × 10^10^ is four times lower than DBO’s 2.21 × 10^11^, cutting optimization error by 74.7%. Its standard deviation 2.22 × 10^10^ is far smaller than GWO’s 1.16 × 10^10^, reflecting stable search without divergence. Original HLOA, DBO and WOA converge slowly with high final fitness, lacking adaptive directional adjustment under huge decision spaces. On multimodal functions such as F9, HSHLOA average fitness at 25,500 reduces HLOA’s 52,100 by 51.1%, and its standard deviation is less than half of DBO’s. Most competitors get trapped halfway with flat curves, but homogenized chaotic initialization scatters initial points evenly. Iterative lens imaging opposite solutions continuously generate new directions to avoid premature convergence, yielding much smaller fluctuation across 30 runs. For hybrid and composite functions (e.g., F12), HSHLOA’s average 4.98 × 10^9^ is two orders of magnitude lower than DBO’s 1.28 × 10^11^, lowering error by 96.1%. Its standard deviation stays at a tiny level while peers show drastically enlarged volatility. The three tactics cooperate well: chaotic mapping ensures full-space coverage, adaptive guidance locks optimal search directions, and lens imaging breaks dense local traps in twisted high-dimensional terrains.

HSHLOA’s strong high-dimensional adaptability comes from synergy of three improved modules. Homogenized Logistic mapping avoids individual clustering in high dimensions, fixing the defect of random initialization. Nonlinear adaptive factors prevent aimless wandering and speed up convergence. Dynamic lens scaling factors keep injecting novel candidate points to suppress late stagnation. Contrast data clearly verify that other seven algorithms degrade sharply, yet the proposed hybrid framework strongly resists high-dimensional performance loss.

Synthesizing 30D and 100D experimental outcomes, HSHLOA comprehensively improves seven key indicators: convergence speed, precision, global search, local mining, escape ability, stability and high-dimensional adaptability. On all four function categories under two dimension settings, HSHLOA obtains better best, average and std values with statistical support, fully verifying the independent effectiveness and synergistic benefits of our multi-strategy improvements.

### 4.3. Practical Constrained Engineering Optimization

To further verify the practicality, reliability, and constraint handling capability of the Hybrid Strategy Hybrid Optimization Algorithm (HSHLOA) proposed in this paper in real engineering scenarios, it was applied to internationally recognized typical constrained nonlinear engineering optimization problems, namely reinforced concrete beam design, three-bar truss design, and pressure vessel design. These problems are widely used in fields such as mechanical design, civil engineering, and structural mechanics, with the optimization objective of minimizing structural mass or manufacturing cost. They also contain multiple mandatory constraints such as stress constraints, displacement constraints, geometric size constraints, strength constraints, and performance constraints. These problems exhibit strong variable coupling, narrow feasible regions, high degrees of nonlinearity, and dense local optima, which are highly consistent with the problems faced in real engineering design.

#### 4.3.1. Reinforced Concrete Beam Design

The design optimization of reinforced concrete beams represents a highly representative complex constrained optimization problem in the field of civil engineering. The optimization objective is to minimize material costs, with decision variables including section width, section height, tensile reinforcement area, etc. The constraints encompass multiple strong constraints such as flexural capacity, shear capacity, deflection limits, crack width, reinforcement ratio, geometric dimensions, etc. The feasible region is extremely narrow, demanding extremely high global search capabilities and constraint handling abilities from the algorithm. Assuming a simply supported beam span of 30 ft, with simply supported supports, bearing a dead load of 1000 lb (including self-weight) and a live load of 2000lb; concrete compressive strength *σ_c_* = 5 ksi; steel yield strength *σ_y_* = 50 ksi. Concrete unit price: 0.02 USD/square meter/linear foot; steel unit price: 1.0 USD/square meter/linear foot. Design variables: To minimize the total cost of the structure to the greatest extent, it is necessary to determine the reinforcement area *A_s_* (=*x*_1_), beam width *b* (=*x*_2_), and beam depth *h* (=*x*_3_). According to ACI Building Code 318-77, the structure should have the required strength proportionally.

(16) $$\begin{array}{c} M_{u}=0.9A_{s}\sigma_{y}\left(0.8h\right)\left(1.0-0.59\frac{A_{s}\sigma_{y}}{0.8bh\sigma_{c}}\right)\geq 1.4M_{d}+1.7M_{l} \end{array}$$

where *M_u_*, *M_d_*, and *M_l_* represent the bending strength, dead load, and live load moment of the beam, respectively. In this case, *M_d_* = 1350 (unit: kip) and *M_l_* = 2700 (unit: kip). The ratio of the depth to the width of the beam is limited to be less than or equal to 4. The optimization problem can be expressed as follows:

Objective function (minimization of total cost):

(17) $$\begin{array}{c} \min f\left(X\right)=2.9x_{1}+0.6x_{2}x_{3} \end{array}$$

Constraint:

Limit value of beam height-to-width ratio:

(18) $$\begin{array}{c} g_{1}\left(X\right)=\frac{x_{2}}{x_{3}}-4\leq 0 \end{array}$$

Equivalent constraint of flexural bearing capacity:

(19) $$\begin{array}{c} g_{2}\left(X\right)=180+7.375\frac{x_{1}^{2}}{x_{3}}-x_{1}x_{2}\leq 0 \end{array}$$

**Figure 6:**
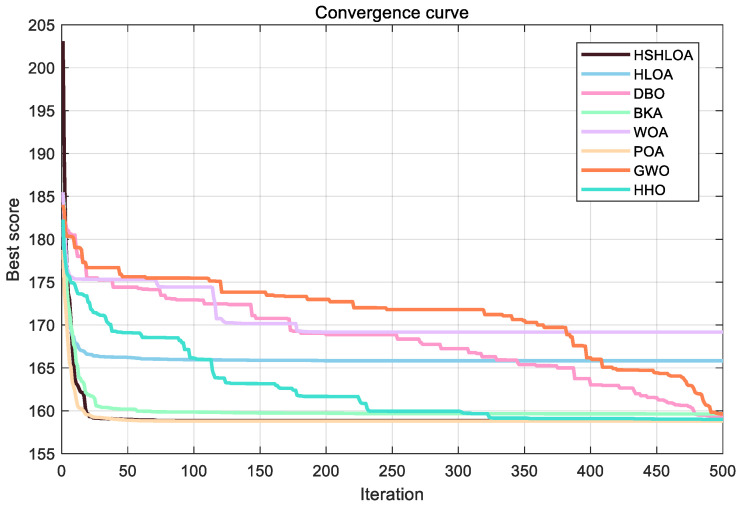
Comparison of average convergence curves for reinforced concrete beam design.

Boundary constraints: $x_{1}\in \left\{6,\;6.16,\;6.32,\;6.6,\;7,\;7.11,\;7.2,\;7.8,\;7.9,\;8,\;8.4\right\}$, $x_{2}\in \left\{28,29,30,\ldots ,40\right\}$, $5\leq x_{3}\leq 10$. The design results of reinforced concrete beams obtained using eight optimization algorithms are shown in Figure 6. The comparison of design performance and simulation time for reinforced concrete beams are presented in Table 7.

The results indicate that HSHLOA can stably meet all constraint conditions during the iterative process, consistently outputting fully feasible high-quality design schemes, with its optimization cost significantly lower than that of the seven algorithms: HLOA, DBO, BKA, WOA, POA, GWO, and HHO. Simultaneously, HSHLOA converges faster, finding the optimal solution within fewer iterations, and exhibits extremely high consistency in multiple runs, with stability far superior to other algorithms. The worst-case cost of the original HLOA increased by 15.1% compared to the optimal cost, and the average cost was 4.4% higher than that of HSHLO. The average cost of WOA increased by 6.3%, with a single worst-case cost increase of 13.8%. The average costs of BKA and GWO were 0.6% and 0.5% higher than the optimal values, respectively. In terms of stability, the fluctuation range of the original HLOA data was over 50,000 times that of HSHLO, and the dispersion index of WOA improved by over 40,000 times compared to the algorithm in this paper. The algorithm’s running time increased only slightly, within the tolerance range of conventional engineering calculations. The adaptive optimal guidance can dynamically shrink the search step size at narrow constraint boundaries, precisely locking in the optimal size; lens imaging learning avoids a large number of local sub-optimal solutions within the constrained space, stably achieving optimal cost while meeting all specification constraints, making it highly suitable for the optimization design of large quantities of civil engineering components.

#### 4.3.2. Three-Bar Truss Design

The design optimization of a three-bar truss is a classic benchmark problem in the field of structural mechanics. The objective is to minimize the structural volume, with constraints on stress and displacement. The problem exhibits significant non-convexity and a large number of local optima, making the algorithm prone to premature convergence. The objective function and constraint conditions are as follows:

Objective function:

(20) $$\min f(x)=\left(2\sqrt{2}x_{1}+x_{2}\right)\times l$$

Constraints:

(21) $$g_{1}(x)=\frac{\sqrt{2}x_{1}+x_{2}}{\sqrt{2}x_{1}^{2}+2x_{1}x_{2}}P-\sigma \leq 0$$

(22) $$g_{2}(x)=\frac{x_{2}}{\sqrt{2}x_{1}^{2}+2x_{1}x_{2}}P-\sigma \leq 0$$

(23) $$g_{3}(x)=\frac{1}{\sqrt{2}x_{2}+x_{1}}P-\sigma \leq 0$$

$0\leq x_{i}\leq 1,\;i=1,2$.

Boundary constraints: *l* = 100 cm, *P* = 2 kN/cm^2^, *σ* = 2 kN/cm^2^.

**Figure 7:**
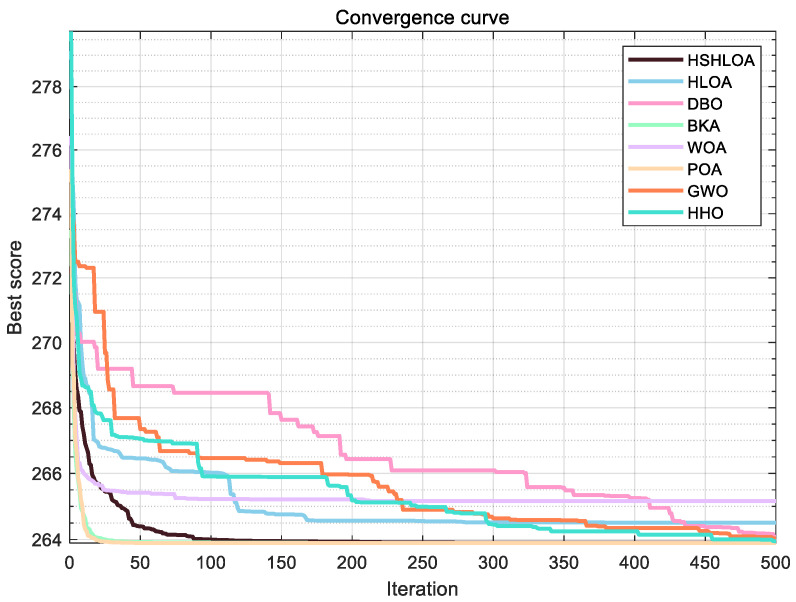
Comparison of convergence curves for three-bar truss structure.

The optimization results of the three-bar truss structure obtained using eight optimization algorithms are shown in Figure 7. The comparison of optimization performance and simulation time for the three-bar truss structure are presented in Table 8.

The results indicate that HSHLOA can stably and reliably converge to the global optimum solution, with a significantly better structural volume optimization effect compared to all comparative algorithms, and fully meeting all mechanical performance constraints. The average optimization result of the original HLOA is 0.37% higher than that of HSHLO, and the worst single volume is 2.2% higher than the optimal design; the average result of WO is 0.34% higher than that of the algorithm in this paper, and the volume increase of the worst scheme is close to 1.5%. In terms of stability, the fluctuation range of the original HLOA result is 590 times that of HSHLOA, the dispersion degree of WO data is about 470 times that of HSHLO, and the fluctuation range of GWO result is 83 times higher compared to the algorithm in this paper. All 30 experimental results of HSHLOA converge completely to the optimal design without any inferior solutions, with an average stability improvement of over 85% compared to other algorithms. In terms of computational cost, HSHLO only takes 17% more time than the original HLO, achieving 100% usable design schemes with a small time investment, highlighting engineering economy. Mechanistically, homogenization chaos initialization enables initial individuals to uniformly cover the feasible region, significantly reducing the probability of initial solutions falling into local optima; lens imaging learning continuously generates inverse candidate solutions, breaking the population aggregation trend throughout the iteration process. Therefore, in truss lightweight design, HSHLO does not require multiple repeated calculations and directly outputs reliable structural dimensions, which can shorten the structural design cycle.

#### 4.3.3. Pressure Vessel Design

Pressure vessel design optimization stands as the most representative complex constrained engineering optimization problem in the field of mechanical engineering. The objective is to minimize manufacturing costs, with decision variables encompassing shell thickness, head thickness, inner diameter, and length. The constraints involve multiple nonlinear constraints such as strength, volume, geometric dimensions, and safety standards. The feasible region is extremely narrow, and the constraint gradients are complex, making it the “gold standard” for testing the optimization capabilities of algorithms in engineering. The goal of pressure vessel design is to minimize the total cost *f*(*x*) while meeting production needs. This problem involves four design variables: shell thickness *T_s_* (corresponding to design variable *x*_3_), head thickness *T_h_* (corresponding to design variable *x*_4_), both of which are integer multiples of 0.0625, inner radius *R* (corresponding to design variable *x*_1_), and vessel length *L* (corresponding to design variable *x*_2_, excluding the head): both are continuous variables.

Objective function:

(24) $$\min f(x)=0.622,4x_{1}x_{3}x_{4}+1.778,1x_{2}x_{3}^{2}+3.166,1x_{1}^{2}x_{4}+19.84x_{1}^{2}x_{3}$$

(25) $$g_{1}(x)=-x_{1}+0.019,3x_{3}⩽0$$

(26) $$g_{2}(x)=-x_{2}+0.009,54x_{3}⩽0$$

Constraints:

(27) $$g_{3}(x)=-\pi x_{3}^{2}x_{4}-\frac{4}{3}\pi x_{3}^{3}+1,296,000⩽0$$

(28) $$g_{4}(x)=x_{4}-240⩽0$$

Boundary constraints: $0⩽x_{1}⩽99,0⩽x_{2}⩽99,10⩽x_{3}⩽200,10⩽x_{4}⩽200$.

**Figure 8:**
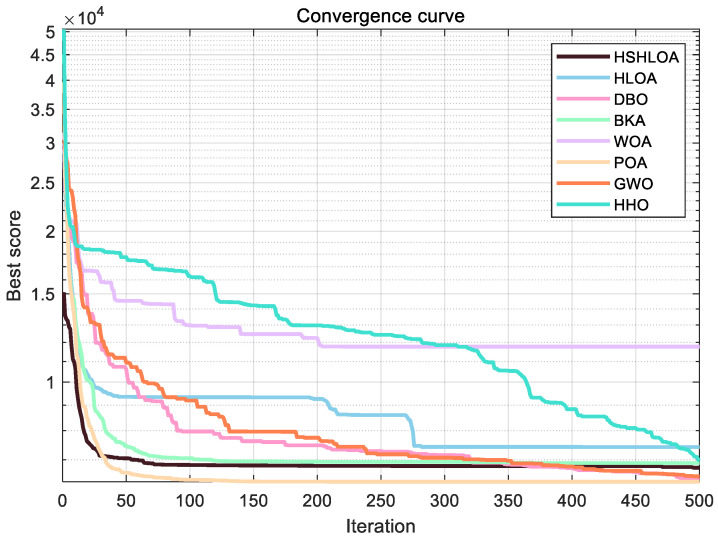
Comparison of average convergence curves in pressure vessel design.

The results of pressure vessel design obtained using eight optimization algorithms are shown in Figure 8. The comparison of pressure vessel design performance and simulation time are presented in Table 9.

The results indicate that HSHLOA can efficiently handle highly nonlinear constraints, achieving a constraint satisfaction rate of 100% and never outputting infeasible solutions. Its manufacturing cost is significantly lower than that of the other seven comparative algorithms, with a stable and outstanding optimization effect. The average manufacturing cost of the original HLOA is 9.9% higher than that of HSHLO, with a single worst-case cost increase of 45.9%. The average cost of WOA increases by 74.8%, and the worst-case manufacturing cost is 182.5% higher. The average costs of BKA and HHO increase by 2.2% and 3.4%, respectively. In terms of stability indicators, the fluctuation range of the original HLOA is 3.75 times that of HSHLO, and the stability index of WO is 13 times that of the algorithms in this paper. Although DBO and GWO have slightly better optimal data, their worst-case manufacturing costs are about 8.8% and 8.8% higher than that of HSHLO, respectively. Relying on a small number of occasional high-quality solutions to depress the average value, the actual engineering reuse risk is high. HSHLOA has moderate computational efficiency, with chaotic initialization broadening the initial search range, avoiding individuals falling into infeasible areas, and adaptively guiding rapid convergence to high-quality feasible regions. Lens imaging continuously breaks through the local optimum trap brought by constraints. The comprehensive performance is improved by an average of 11% compared to traditional algorithms, making it more suitable for standardized batch design of pressure vessels.

Based on the application results of three typical constrained engineering optimization problems, it can be concluded that the HSHLOA proposed in this paper possesses comprehensive and significant advantages in engineering scenarios. Firstly, HSHLOA exhibits extremely strong constraint handling capabilities, consistently meeting various constraints such as stress, displacement, strength, geometry, and performance, and always producing fully feasible engineering solutions. Secondly, HSHLOA boasts the highest optimization accuracy, achieving optimal levels in engineering indicators such as cost, quality, and volume, with significantly better optimization results than existing mainstream algorithms. Thirdly, HSHLOA converges faster and has higher iteration efficiency, significantly reducing engineering optimization calculation time and enhancing design efficiency. Fourthly, HSHLOA demonstrates the best numerical stability, with minimal fluctuation in multiple runs, producing reliable and repeatable output results that fully meet the practical requirements of engineering applications. Fifthly, HSHLOA possesses strong versatility, adapting to optimization problems in different fields such as civil engineering, mechanical structure, and equipment design, exhibiting broad engineering applicability. Experimental results fully demonstrate that the multi-strategy hybrid improvement framework proposed in this paper not only significantly enhances the algorithm’s performance on standard test functions but also effectively translates into practical optimization capabilities in engineering scenarios, making HSHLOA an efficient, reliable, and practical engineering optimization tool.

## 5. Conclusions

This paper proposes a Hybrid Strategy Hybridized Optimization Algorithm (HSHLOA) to address a series of core deficiencies in the original Horned Lizards Optimization Algorithm (HLOA), including uneven population initialization distribution, imbalance between global exploration and local exploitation, tendency to trap in local optima in the later stages of iteration, insufficient convergence accuracy, significant performance degradation under high-dimensional conditions, and poor numerical stability. The algorithm enhances the uniformity, ergodicity, and diversity of the initial population by introducing a homogenized Logistic chaotic mapping, thereby improving algorithm performance from the starting point of search. It achieves dynamic balance between global exploration and local exploitation during the iteration process by constructing an adaptive optimal guidance strategy, significantly accelerating convergence speed and improving convergence accuracy. By embedding a lens imaging learning strategy to generate dynamic adaptive inverse solutions, an efficient local optimum escape mechanism is constructed, fundamentally alleviating the premature convergence problem. The three improvement strategies complement each other and synergize, achieving systematic improvement in the global optimization performance of the Horned Lizards Optimization Algorithm without increasing the time complexity of the algorithm. Comparative experiments based on the CEC 2017 test set under 30-dimensional and 100-dimensional conditions show that HSHLOA is significantly superior to the original HLOA, DBO, BKA, WOA, POA, GWO, and HHO, seven mainstream swarm intelligence optimization algorithms, in terms of convergence speed, convergence accuracy, global exploration, local exploitation, local optimum escape, numerical stability, and high-dimensional adaptability. Further application results in four typical constrained engineering optimization problems verify that HSHLOA possesses excellent constraint handling capabilities, high-precision optimization capabilities, and engineering practicality, providing efficient and reliable solutions for complex engineering optimization problems.

Future research will expand in multiple directions based on existing work. Firstly, the HSHLOA will be extended to multi-objective optimization scenarios, constructing a multi-objective optimization version that can simultaneously meet multiple indicators such as cost, quality, performance, and safety, to adapt to more complex engineering decision-making needs. Secondly, adaptive mechanisms and reinforcement learning strategies will be further combined to enhance the algorithm’s optimization performance in dynamic environments, noise interference, and large-scale data conditions, strengthening the algorithm’s environmental adaptability and robustness. Apart from the civil and mechanical structural optimization cases adopted in this paper, we further analyze the inherent adaptability and unique superiority of HSHLOA for a series of non-mechanical and civil engineering optimization tasks, including intelligent equipment parameter identification, new energy system dispatching, aerospace aerodynamic layout and path planning, wireless sensor network deployment, as well as deep learning hyperparameter tuning and biomedical image segmentation. Finally, in-depth theoretical analysis of the convergence theory, time complexity proof, and performance boundary analysis of HSHLOA will be conducted to improve the theoretical foundation system of the algorithm, providing more solid theoretical support for further optimization and wide application of the algorithm.

## Figures and Tables

**Table 1:** The result of standard functions of CEC2017 (Dim = 30).

		HSHLOA	HLOA	DBO	BKA	WOA	POA	GWO	HHO
F1	min	6.77E+02	8.97E+06	2.37E+10	1.04E+08	6.79E+08	9.24E+09	2.16E+10	1.47E+07
F1	std	1.39E+05	3.65E+07	4.04E+09	1.01E+10	7.81E+08	5.29E+09	5.14E+09	9.04E+06
F1	avg	3.82E+04	4.54E+07	2.88E+10	5.21E+09	1.65E+09	1.65E+10	2.86E+10	3.17E+07
F1	median	5.53E+03	3.14E+07	2.77E+10	2.39E+09	1.55E+09	1.55E+10	2.72E+10	2.97E+07
F3	min	1.49E+04	9.15E+03	6.19E+04	5.11E+03	1.64E+05	2.11E+04	5.56E+04	2.36E+04
F3	std	8.65E+03	1.05E+04	7.18E+03	2.36E+04	5.66E+04	6.95E+03	8.46E+03	7.24E+03
F3	avg	3.09E+04	2.36E+04	7.76E+04	2.46E+04	2.40E+05	3.26E+04	7.50E+04	3.87E+04
F3	median	2.88E+04	2.31E+04	7.85E+04	1.68E+04	2.29E+05	3.28E+04	7.59E+04	3.86E+04
F4	min	4.20E+02	4.92E+02	2.18E+03	4.74E+02	6.94E+02	5.75E+02	3.19E+03	5.02E+02
F4	std	3.56E+01	3.90E+01	2.36E+03	2.88E+03	1.31E+02	1.07E+03	2.07E+03	4.01E+01
F4	avg	4.98E+02	5.48E+02	6.21E+03	2.09E+03	8.49E+02	1.78E+03	6.35E+03	5.64E+02
F4	median	4.91E+02	5.46E+02	5.91E+03	6.82E+02	8.36E+02	1.37E+03	6.37E+03	5.59E+02
F5	min	6.36E+02	7.09E+02	7.75E+02	6.45E+02	7.38E+02	6.85E+02	7.51E+02	6.90E+02
F5	std	4.71E+01	5.09E+01	2.79E+01	7.10E+01	5.41E+01	3.41E+01	3.60E+01	3.41E+01
F5	avg	7.37E+02	7.91E+02	8.30E+02	7.56E+02	8.56E+02	7.56E+02	8.22E+02	7.54E+02
F5	median	7.46E+02	7.81E+02	8.34E+02	7.32E+02	8.59E+02	7.70E+02	8.14E+02	7.50E+02
F6	min	6.29E+02	6.55E+02	6.55E+02	6.49E+02	6.52E+02	6.39E+02	6.56E+02	6.48E+02
F6	std	8.22E+00	7.89E+00	4.92E+00	6.61E+00	1.14E+01	7.57E+00	5.49E+00	7.54E+00
F6	avg	6.52E+02	6.74E+02	6.67E+02	6.60E+02	6.80E+02	6.58E+02	6.69E+02	6.65E+02
F6	median	6.53E+02	6.74E+02	6.67E+02	6.61E+02	6.80E+02	6.58E+02	6.70E+02	6.66E+02
F7	min	1.05E+03	1.19E+03	1.16E+03	1.11E+03	1.12E+03	1.13E+03	1.09E+03	1.14E+03
F7	std	8.00E+01	7.22E+01	4.81E+01	5.81E+01	8.74E+01	7.13E+01	4.89E+01	6.46E+01
F7	avg	1.23E+03	1.31E+03	1.22E+03	1.23E+03	1.31E+03	1.25E+03	1.21E+03	1.29E+03
F7	median	1.26E+03	1.31E+03	1.21E+03	1.25E+03	1.33E+03	1.26E+03	1.21E+03	1.29E+03
F8	min	8.95E+02	9.50E+02	9.97E+02	8.98E+02	9.92E+02	9.37E+02	1.02E+03	9.41E+02
F8	std	3.63E+01	3.06E+01	2.48E+01	5.33E+01	4.22E+01	2.40E+01	2.12E+01	1.85E+01
F8	avg	9.76E+02	1.01E+03	1.06E+03	9.87E+02	1.05E+03	9.93E+02	1.06E+03	9.75E+02
F8	median	9.80E+02	1.01E+03	1.06E+03	9.76E+02	1.03E+03	9.98E+02	1.06E+03	9.73E+02
F9	min	2.74E+03	5.39E+03	6.13E+03	4.31E+03	5.30E+03	4.20E+03	5.66E+03	5.66E+03
F9	std	7.04E+02	9.43E+02	1.20E+03	1.86E+03	3.74E+03	7.25E+02	1.34E+03	8.28E+02
F9	avg	4.55E+03	6.64E+03	7.88E+03	5.84E+03	1.03E+04	5.57E+03	7.92E+03	8.05E+03
F9	median	4.52E+03	6.64E+03	7.79E+03	5.28E+03	9.39E+03	5.68E+03	7.79E+03	8.06E+03
F10	min	3.38E+03	4.87E+03	6.03E+03	4.31E+03	5.68E+03	3.75E+03	6.62E+03	4.98E+03
F10	std	1.23E+03	8.99E+02	7.29E+02	6.42E+02	6.88E+02	6.90E+02	6.84E+02	6.88E+02
F10	avg	5.49E+03	6.70E+03	8.03E+03	5.19E+03	7.18E+03	5.22E+03	7.98E+03	5.90E+03
F10	median	5.18E+03	6.69E+03	8.22E+03	5.06E+03	7.19E+03	5.29E+03	7.81E+03	5.70E+03
F11	min	1.23E+03	1.32E+03	3.46E+03	1.29E+03	2.01E+03	1.51E+03	4.29E+03	1.30E+03
F11	std	6.34E+01	3.09E+02	1.90E+03	4.23E+03	2.76E+03	1.21E+03	1.85E+03	1.16E+02
F11	avg	1.35E+03	1.60E+03	6.90E+03	2.41E+03	5.03E+03	2.82E+03	7.35E+03	1.45E+03
F11	median	1.34E+03	1.51E+03	7.09E+03	1.40E+03	4.12E+03	2.41E+03	7.16E+03	1.43E+03
F12	min	1.34E+05	2.10E+06	2.03E+09	8.99E+05	3.68E+07	2.81E+07	1.14E+09	4.04E+06
F12	std	8.05E+05	1.90E+07	2.33E+09	2.58E+09	1.84E+08	1.28E+09	1.87E+09	1.62E+07
F12	avg	1.13E+06	2.10E+07	6.02E+09	6.54E+08	2.37E+08	1.38E+09	5.98E+09	2.44E+07
F12	median	7.91E+05	1.75E+07	6.39E+09	8.44E+06	2.35E+08	1.30E+09	6.09E+09	2.18E+07
F13	min	4.08E+03	2.73E+04	8.79E+07	7.15E+04	2.12E+05	2.83E+04	4.87E+08	2.63E+05
F13	std	1.31E+07	1.44E+06	1.88E+09	2.85E+08	2.24E+06	5.30E+07	1.51E+09	6.61E+05
F13	avg	2.73E+06	5.84E+05	1.97E+09	9.71E+07	2.43E+06	3.07E+07	2.68E+09	7.61E+05
F13	median	3.40E+04	9.88E+04	1.20E+09	1.60E+05	1.90E+06	1.09E+06	2.22E+09	5.86E+05
F14	min	1.45E+04	2.94E+03	2.60E+04	1.68E+03	4.79E+04	1.77E+03	3.92E+04	4.86E+04
F14	std	5.88E+04	8.96E+04	4.87E+05	6.47E+04	1.89E+06	3.12E+04	4.48E+05	6.03E+05
F14	avg	7.95E+04	6.58E+04	4.88E+05	2.55E+04	1.73E+06	2.18E+04	6.40E+05	6.25E+05
F14	median	6.22E+04	3.41E+04	2.66E+05	3.83E+03	1.38E+06	8.31E+03	5.51E+05	5.42E+05
F15	min	2.20E+03	2.83E+03	3.45E+05	9.70E+03	5.93E+04	1.24E+04	1.78E+05	1.64E+04
F15	std	1.01E+04	3.72E+04	4.00E+06	1.52E+05	4.17E+06	5.94E+04	2.67E+06	4.72E+04
F15	avg	1.05E+04	2.80E+04	4.20E+06	7.14E+04	2.33E+06	5.08E+04	3.11E+06	7.97E+04
F15	median	6.94E+03	1.66E+04	2.52E+06	3.41E+04	1.05E+06	3.14E+04	2.39E+06	7.05E+04
F16	min	2.14E+03	2.69E+03	2.86E+03	2.46E+03	3.28E+03	2.42E+03	3.20E+03	2.80E+03
F16	std	3.75E+02	8.00E+02	4.24E+02	4.99E+02	5.91E+02	5.16E+02	3.23E+02	3.75E+02
F16	avg	2.85E+03	3.57E+03	3.88E+03	3.13E+03	4.19E+03	3.17E+03	3.82E+03	3.39E+03
F16	median	2.78E+03	3.42E+03	3.84E+03	3.00E+03	4.11E+03	3.06E+03	3.87E+03	3.41E+03
F17	min	2.07E+03	2.39E+03	2.18E+03	1.98E+03	2.12E+03	1.89E+03	2.33E+03	2.10E+03
F17	std	2.56E+02	3.47E+02	2.90E+02	2.50E+02	2.69E+02	2.08E+02	3.37E+02	2.60E+02
F17	avg	2.54E+03	2.91E+03	2.80E+03	2.39E+03	2.77E+03	2.26E+03	2.74E+03	2.60E+03
F17	median	2.58E+03	2.83E+03	2.79E+03	2.38E+03	2.77E+03	2.21E+03	2.68E+03	2.62E+03
F18	min	2.42E+04	8.41E+04	2.18E+05	1.82E+04	3.49E+05	4.24E+04	3.77E+05	5.09E+04
F18	std	2.77E+05	8.24E+05	4.26E+06	8.95E+04	1.13E+07	1.37E+05	3.90E+06	2.89E+06
F18	avg	2.77E+05	7.23E+05	4.96E+06	1.06E+05	9.42E+06	1.84E+05	3.63E+06	1.74E+06
F18	median	1.62E+05	4.53E+05	3.46E+06	8.18E+04	5.93E+06	1.48E+05	2.29E+06	7.41E+05
F19	min	1.96E+03	3.08E+03	4.63E+07	9.66E+03	9.08E+05	2.12E+04	1.11E+07	5.71E+04
F19	std	2.02E+03	1.23E+05	2.66E+08	3.72E+06	1.90E+07	7.46E+05	1.71E+08	7.22E+05
F19	avg	4.42E+03	9.22E+04	2.81E+08	8.44E+05	1.73E+07	8.04E+05	2.17E+08	9.72E+05
F19	median	3.94E+03	3.31E+04	2.35E+08	1.18E+05	8.93E+06	4.88E+05	1.52E+08	7.56E+05
F20	min	2.26E+03	2.47E+03	2.38E+03	2.23E+03	2.30E+03	2.27E+03	2.36E+03	2.45E+03
F20	std	2.24E+02	2.59E+02	1.63E+02	1.68E+02	2.13E+02	1.46E+02	1.59E+02	2.26E+02
F20	avg	2.78E+03	3.04E+03	2.66E+03	2.54E+03	2.84E+03	2.48E+03	2.70E+03	2.80E+03
F20	median	2.83E+03	3.05E+03	2.64E+03	2.55E+03	2.81E+03	2.46E+03	2.70E+03	2.77E+03
F21	min	2.20E+03	2.26E+03	2.69E+03	2.25E+03	2.33E+03	2.34E+03	2.83E+03	2.31E+03
F21	std	9.93E+02	8.75E+02	3.35E+02	1.12E+03	9.65E+02	9.45E+02	4.65E+02	6.38E+02
F21	avg	3.24E+03	4.41E+03	3.26E+03	3.67E+03	4.83E+03	3.66E+03	3.32E+03	4.16E+03
F21	median	3.78E+03	4.72E+03	3.17E+03	4.14E+03	4.82E+03	4.00E+03	3.15E+03	4.38E+03
F22	min	9.57E+03	1.27E+04	1.48E+04	1.09E+04	1.31E+04	9.92E+03	1.50E+04	1.13E+04
F22	std	2.05E+03	2.58E+03	2.23E+03	1.91E+03	2.02E+03	1.66E+03	1.98E+03	2.18E+03
F22	avg	1.51E+04	1.78E+04	2.07E+04	1.41E+04	1.85E+04	1.32E+04	2.07E+04	1.60E+04
F22	median	1.47E+04	1.77E+04	2.16E+04	1.39E+04	1.88E+04	1.33E+04	2.11E+04	1.61E+04
F23	min	4.10E+03	5.84E+03	5.46E+03	5.08E+03	3.70E+03	5.10E+03	5.37E+03	5.95E+03
F23	std	8.00E+02	1.19E+03	5.24E+02	7.81E+02	9.79E+02	6.02E+02	5.66E+02	8.59E+02
F23	avg	5.51E+03	7.76E+03	6.36E+03	6.68E+03	6.77E+03	6.19E+03	6.33E+03	7.47E+03
F23	median	5.39E+03	7.73E+03	6.33E+03	6.51E+03	6.89E+03	6.17E+03	6.20E+03	7.53E+03
F24	min	2.50E+03	2.62E+03	4.59E+03	3.36E+03	2.76E+03	3.94E+03	4.39E+03	5.17E+03
F24	std	1.32E+03	6.50E+02	5.55E+02	8.21E+02	7.17E+02	5.20E+02	5.00E+02	2.01E+02
F24	avg	5.15E+03	5.71E+03	5.91E+03	5.40E+03	5.89E+03	5.55E+03	6.05E+03	5.86E+03
F24	median	5.77E+03	5.79E+03	6.14E+03	5.67E+03	5.91E+03	5.70E+03	6.21E+03	5.87E+03
F25	min	2.88E+03	2.90E+03	3.64E+03	2.92E+03	2.94E+03	3.00E+03	3.46E+03	2.89E+03
F25	std	1.25E+01	3.01E+01	2.55E+02	1.72E+02	4.87E+01	1.56E+02	3.66E+02	2.25E+01
F25	avg	2.89E+03	2.96E+03	4.07E+03	3.03E+03	3.08E+03	3.20E+03	4.09E+03	2.93E+03
F25	median	2.88E+03	2.96E+03	4.05E+03	2.99E+03	3.07E+03	3.15E+03	4.12E+03	2.93E+03
F26	min	2.90E+03	3.28E+03	6.40E+03	4.67E+03	6.58E+03	4.81E+03	4.86E+03	3.33E+03
F26	std	1.66E+03	1.46E+03	6.50E+02	1.39E+03	1.07E+03	1.37E+03	9.18E+02	1.48E+03
F26	avg	6.76E+03	9.50E+03	7.49E+03	7.99E+03	8.25E+03	7.23E+03	7.64E+03	7.62E+03
F26	median	7.01E+03	9.57E+03	7.52E+03	8.05E+03	8.31E+03	7.43E+03	7.62E+03	7.95E+03
F27	min	3.21E+03	3.32E+03	3.42E+03	3.24E+03	3.27E+03	3.24E+03	3.34E+03	3.31E+03
F27	std	1.59E+02	3.71E+02	1.55E+02	1.68E+02	1.20E+02	7.62E+01	1.53E+02	1.68E+02
F27	avg	3.41E+03	3.67E+03	3.65E+03	3.41E+03	3.46E+03	3.39E+03	3.63E+03	3.47E+03
F27	median	3.38E+03	3.59E+03	3.63E+03	3.35E+03	3.45E+03	3.39E+03	3.62E+03	3.40E+03
F28	min	3.20E+03	3.26E+03	4.09E+03	3.22E+03	3.43E+03	3.49E+03	4.68E+03	3.27E+03
F28	std	4.48E+01	2.96E+01	4.37E+02	1.33E+03	1.34E+02	4.25E+02	3.26E+02	2.74E+01
F28	avg	3.24E+03	3.31E+03	5.11E+03	3.95E+03	3.61E+03	3.97E+03	5.20E+03	3.33E+03
F28	median	3.23E+03	3.31E+03	5.04E+03	3.40E+03	3.59E+03	3.82E+03	5.17E+03	3.33E+03
F29	min	3.62E+03	4.82E+03	4.31E+03	4.03E+03	4.26E+03	4.05E+03	4.33E+03	3.94E+03
F29	std	3.15E+02	7.13E+02	2.98E+02	3.06E+02	5.43E+02	3.47E+02	2.89E+02	3.78E+02
F29	avg	4.24E+03	5.91E+03	4.91E+03	4.56E+03	5.34E+03	4.66E+03	4.80E+03	4.63E+03
F29	median	4.17E+03	5.81E+03	4.96E+03	4.57E+03	5.32E+03	4.74E+03	4.74E+03	4.58E+03
F30	min	6.83E+03	6.06E+04	1.55E+07	3.17E+05	2.29E+06	8.98E+05	9.64E+06	4.32E+05
F30	std	3.88E+06	1.75E+08	1.03E+08	2.38E+07	4.30E+07	9.50E+06	2.36E+08	2.94E+06
F30	avg	7.26E+05	3.32E+07	1.12E+08	6.60E+06	4.52E+07	9.00E+06	1.25E+08	4.26E+06
F30	median	1.64E+04	8.90E+05	6.04E+07	1.82E+06	2.88E+07	7.95E+06	7.45E+07	3.30E+06

**Table 2:** Ranking matrix of 8 algorithms CEC2017 (Dim = 30).

Function	HSHLOA	HLOA	DBO	BKA	WOA	POA	GWO	HHO
F1	1	3	8	5	4	6	7	2
F3	3	1	7	2	8	4	6	5
F4	1	2	7	6	4	5	8	3
F5	1	5	7	4	8	3	6	2
F6	1	7	5	3	8	2	6	4
F7	4	8	2	3	7	5	1	6
F8	2	5	8	3	6	4	7	1
F9	1	4	5	3	8	2	6	7
F10	3	5	8	1	6	2	7	4
F11	1	3	7	4	6	5	8	2
F12	1	2	8	5	4	6	7	3
F13	4	1	7	6	3	5	8	2
F14	4	3	5	2	8	1	7	6
F15	1	2	8	4	6	3	7	5
F16	1	5	7	2	8	3	6	4
F17	3	8	7	2	6	1	5	4
F18	3	4	7	1	8	2	6	5
F19	1	2	8	4	6	3	7	5
F20	5	8	3	2	7	1	4	6
F21	1	7	2	5	8	4	3	6
F22	3	5	7	2	6	1	8	4
F23	1	8	4	5	6	2	3	7
F24	1	4	7	2	6	3	8	5
F25	1	3	7	4	5	6	8	2
F26	1	8	3	6	7	2	5	4
F27	3	8	7	2	4	1	6	5
F28	1	2	7	5	4	6	8	3
F29	1	8	6	2	7	4	5	3
F30	1	5	7	3	6	4	8	2

**Table 3:** Comparison of Wilcoxon rank-sum results for CEC2017 (Dim = 30).

Function	HSHLOA	HLOA	DBO	BKA	WOA	POA	GWO
F1	3.02E−11	3.02E−11	3.02E−11	3.02E−11	3.02E−11	3.02E−11	3.02E−11
F3	4.86E−03	3.02E−11	1.34E−05	3.02E−11	2.97E−01	3.02E−11	5.56E−04
F4	1.29E−06	3.02E−11	1.86E−09	3.02E−11	4.08E−11	3.02E−11	4.69E−08
F5	9.03E−04	2.37E−10	7.17E−01	2.67E−09	1.19E−01	1.17E−09	3.26E−01
F6	2.37E−10	1.55E−09	2.25E−04	5.07E−10	5.32E−03	6.72E−10	1.36E−07
F7	5.56E−04	1.86E−01	7.06E−01	3.77E−04	7.17E−01	3.92E−02	1.22E−02
F8	1.86E−03	2.37E−10	8.77E−01	3.65E−08	5.55E−02	1.21E−10	6.52E−01
F9	5.49E−11	3.02E−11	2.13E−04	8.15E−11	5.46E−06	3.34E−11	3.34E−11
F10	8.15E−05	1.69E−09	3.71E−01	5.19E−07	5.69E−01	1.86E−09	6.15E−02
F11	6.52E−09	3.02E−11	4.23E−03	3.02E−11	3.34E−11	3.02E−11	2.13E−04
F12	4.98E−11	3.02E−11	8.10E−10	3.02E−11	3.02E−11	3.02E−11	3.02E−11
F13	1.25E−04	3.02E−11	2.57E−07	6.01E−08	5.60E−07	3.02E−11	1.16E−07
F14	4.06E−02	3.09E−06	2.03E−07	4.18E−09	5.19E−07	4.57E−09	3.82E−09
F15	7.30E−04	3.02E−11	1.31E−08	3.02E−11	6.53E−08	3.02E−11	1.33E−10
F16	2.60E−05	4.20E−10	2.32E−02	9.92E−11	1.22E−02	2.15E−10	3.57E−06
F17	3.59E−05	9.03E−04	2.61E−02	2.50E−03	4.08E−05	2.24E−02	4.12E−01
F18	3.34E−03	3.82E−10	5.32E−03	1.46E−10	4.20E−01	4.62E−10	3.59E−05
F19	1.10E−08	3.02E−11	3.69E−11	3.02E−11	3.02E−11	3.02E−11	3.02E−11
F20	2.01E−04	6.97E−03	2.00E−05	5.49E−01	8.20E−07	4.84E−02	9.82E−01
F21	7.09E−08	8.42E−01	1.38E−02	9.26E−09	7.62E−03	9.00E−01	1.17E−05
F22	1.25E−04	7.38E−10	1.84E−02	4.11E−07	6.55E−04	1.96E−10	8.77E−02
F23	2.23E−09	4.74E−06	1.73E−06	2.15E−06	3.01E−04	1.43E−05	2.23E−09
F24	1.37E−01	7.30E−04	4.12E−01	4.64E−03	9.82E−01	2.49E−06	7.29E−03
F25	1.21E−10	3.02E−11	3.69E−11	3.02E−11	3.02E−11	3.02E−11	1.17E−09
F26	2.92E−09	8.77E−02	2.16E−03	2.01E−04	2.46E−01	1.56E−02	2.75E−03
F27	5.61E−05	3.01E−07	6.84E−01	6.35E−02	7.96E−01	2.00E−06	1.02E−01
F28	2.67E−09	3.02E−11	8.89E−10	4.08E−11	3.02E−11	3.02E−11	5.57E−10
F29	4.08E−11	6.52E−09	1.89E−04	9.76E−10	3.16E−05	5.09E−08	1.04E−04
F30	5.07E−10	3.34E−11	5.07E−10	6.70E−11	5.07E−10	4.08E−11	5.57E−10

**Table 4:** The result of standard functions of CEC2017 (Dim = 100).

		HSHLOA	HLOA	DBO	BKA	WOA	POA	GWO	HHO
F1	min	1.66E+10	2.37E+09	2.04E+11	7.02E+10	5.12E+10	1.07E+11	1.91E+11	5.05E+09
F1	std	2.22E+10	1.16E+09	6.86E+09	5.52E+10	8.46E+09	1.94E+10	1.16E+10	1.58E+09
F1	avg	5.59E+10	4.58E+09	2.21E+11	1.28E+11	6.56E+10	1.37E+11	2.22E+11	7.28E+09
F1	median	5.08E+10	4.43E+09	2.22E+11	1.12E+11	6.35E+10	1.37E+11	2.24E+11	6.94E+09
F3	min	2.74E+05	2.42E+05	3.37E+05	1.66E+05	4.60E+05	2.36E+05	3.47E+05	2.79E+05
F3	std	1.82E+04	6.53E+04	1.11E+05	5.39E+04	1.66E+05	1.87E+04	7.64E+04	1.76E+04
F3	avg	3.08E+05	3.32E+05	4.44E+05	2.44E+05	9.09E+05	2.69E+05	4.23E+05	3.15E+05
F3	median	3.06E+05	3.18E+05	4.08E+05	2.31E+05	9.19E+05	2.70E+05	4.09E+05	3.18E+05
F4	min	1.32E+03	1.43E+03	4.58E+04	6.88E+03	7.97E+03	9.46E+03	4.57E+04	1.83E+03
F4	std	9.59E+02	2.94E+02	6.01E+03	2.07E+04	2.17E+03	7.29E+03	6.64E+03	3.90E+02
F4	avg	2.27E+03	1.83E+03	5.73E+04	1.86E+04	1.14E+04	1.91E+04	5.81E+04	2.44E+03
F4	median	1.97E+03	1.79E+03	5.76E+04	1.34E+04	1.09E+04	1.80E+04	5.83E+04	2.45E+03
F5	min	1.29E+03	1.57E+03	1.86E+03	1.34E+03	1.66E+03	1.40E+03	1.85E+03	1.53E+03
F5	std	6.89E+01	7.93E+01	4.76E+01	1.58E+02	1.33E+02	5.46E+01	4.71E+01	4.51E+01
F5	avg	1.40E+03	1.75E+03	1.93E+03	1.49E+03	1.87E+03	1.55E+03	1.94E+03	1.60E+03
F5	median	1.39E+03	1.74E+03	1.93E+03	1.46E+03	1.84E+03	1.55E+03	1.94E+03	1.59E+03
F6	min	6.54E+02	6.81E+02	6.87E+02	6.70E+02	6.87E+02	6.73E+02	6.92E+02	6.82E+02
F6	std	5.09E+00	4.56E+00	4.16E+00	1.21E+01	7.99E+00	3.72E+00	3.62E+00	3.36E+00
F6	avg	6.66E+02	6.92E+02	6.98E+02	6.80E+02	7.03E+02	6.80E+02	6.98E+02	6.87E+02
F6	median	6.66E+02	6.92E+02	6.97E+02	6.76E+02	7.03E+02	6.80E+02	6.98E+02	6.86E+02
F7	min	2.61E+03	3.45E+03	3.42E+03	3.23E+03	3.41E+03	3.21E+03	3.42E+03	3.48E+03
F7	std	1.73E+02	1.37E+02	9.11E+01	1.93E+02	1.57E+02	8.67E+01	1.04E+02	1.23E+02
F7	avg	3.20E+03	3.70E+03	3.63E+03	3.41E+03	3.68E+03	3.45E+03	3.65E+03	3.72E+03
F7	median	3.21E+03	3.71E+03	3.64E+03	3.35E+03	3.66E+03	3.46E+03	3.67E+03	3.72E+03
F8	min	1.61E+03	1.96E+03	2.21E+03	1.79E+03	2.03E+03	1.87E+03	2.15E+03	1.93E+03
F8	std	9.09E+01	9.81E+01	6.53E+01	1.95E+02	1.23E+02	7.30E+01	7.45E+01	7.42E+01
F8	avg	1.83E+03	2.21E+03	2.34E+03	1.98E+03	2.28E+03	2.02E+03	2.33E+03	2.06E+03
F8	median	1.85E+03	2.22E+03	2.36E+03	1.91E+03	2.28E+03	2.03E+03	2.33E+03	2.06E+03
F9	min	1.91E+04	4.59E+04	5.69E+04	2.86E+04	4.92E+04	3.14E+04	6.27E+04	5.06E+04
F9	std	3.66E+03	4.18E+03	7.96E+03	2.67E+03	2.32E+04	2.00E+03	7.42E+03	5.24E+03
F9	avg	2.55E+04	5.21E+04	7.62E+04	3.20E+04	7.59E+04	3.56E+04	7.44E+04	6.39E+04
F9	median	2.53E+04	5.22E+04	7.86E+04	3.09E+04	7.05E+04	3.54E+04	7.22E+04	6.51E+04
F10	min	1.39E+04	2.07E+04	2.69E+04	1.66E+04	2.61E+04	1.57E+04	2.76E+04	2.06E+04
F10	std	2.16E+03	2.32E+03	1.50E+03	3.78E+03	1.39E+03	1.24E+03	1.35E+03	1.28E+03
F10	avg	1.72E+04	2.63E+04	3.12E+04	1.99E+04	2.83E+04	1.92E+04	3.16E+04	2.27E+04
F10	median	1.68E+04	2.64E+04	3.17E+04	1.89E+04	2.81E+04	1.93E+04	3.19E+04	2.27E+04
F11	min	5.22E+04	5.72E+04	1.51E+05	4.72E+04	1.47E+05	5.30E+04	1.58E+05	7.22E+04
F11	std	1.66E+04	1.64E+04	1.48E+04	3.75E+04	1.25E+05	1.81E+04	1.91E+04	1.52E+04
F11	avg	8.77E+04	8.82E+04	1.85E+05	7.25E+04	3.42E+05	9.24E+04	1.99E+05	1.03E+05
F11	median	8.77E+04	8.92E+04	1.86E+05	6.33E+04	3.31E+05	9.38E+04	2.03E+05	1.05E+05
F12	min	6.80E+07	7.84E+08	9.69E+10	8.03E+09	8.04E+09	3.43E+10	1.06E+11	6.66E+08
F12	std	8.58E+09	4.81E+08	1.31E+10	1.08E+10	3.03E+09	2.00E+10	1.39E+10	4.53E+08
F12	avg	4.98E+09	1.68E+09	1.28E+11	2.73E+10	1.20E+10	6.81E+10	1.29E+11	1.32E+09
F12	median	8.19E+08	1.65E+09	1.30E+11	2.67E+10	1.15E+10	6.14E+10	1.26E+11	1.28E+09
F13	min	3.46E+04	2.68E+06	2.23E+10	1.79E+07	1.86E+08	5.07E+09	1.96E+10	9.25E+06
F13	std	5.79E+08	2.17E+07	3.38E+09	1.25E+10	2.87E+08	3.63E+09	4.05E+09	4.46E+06
F13	avg	1.36E+08	2.87E+07	3.13E+10	6.64E+09	5.68E+08	1.35E+10	3.08E+10	1.80E+07
F13	median	6.75E+04	1.94E+07	3.16E+10	2.10E+09	5.03E+08	1.41E+10	3.09E+10	1.77E+07
F14	min	7.97E+05	1.55E+06	1.07E+07	4.09E+05	7.10E+06	1.34E+06	5.89E+06	2.27E+06
F14	std	9.52E+05	2.73E+06	9.42E+06	1.45E+07	5.50E+06	4.32E+06	1.10E+07	2.11E+06
F14	avg	1.90E+06	5.54E+06	2.50E+07	6.73E+06	1.40E+07	7.64E+06	2.39E+07	5.41E+06
F14	median	1.66E+06	5.49E+06	2.33E+07	1.23E+06	1.23E+07	6.96E+06	2.13E+07	5.05E+06
F15	min	5.65E+03	4.37E+05	6.28E+09	1.03E+06	2.49E+07	1.10E+08	5.43E+09	1.91E+06
F15	std	2.70E+04	3.62E+06	1.95E+09	5.95E+09	7.84E+07	3.12E+09	2.23E+09	3.67E+06
F15	avg	3.67E+04	3.97E+06	1.02E+10	2.49E+09	1.04E+08	4.47E+09	9.79E+09	4.79E+06
F15	median	3.10E+04	2.68E+06	1.05E+10	2.23E+07	9.35E+07	4.47E+09	9.78E+09	4.19E+06
F16	min	5.56E+03	7.76E+03	1.31E+04	7.12E+03	1.11E+04	8.75E+03	1.26E+04	6.53E+03
F16	std	7.00E+02	2.67E+03	1.33E+03	3.03E+03	2.42E+03	1.58E+03	1.49E+03	9.75E+02
F16	avg	6.62E+03	1.25E+04	1.58E+04	9.69E+03	1.46E+04	1.12E+04	1.58E+04	8.41E+03
F16	median	6.56E+03	1.22E+04	1.55E+04	8.65E+03	1.42E+04	1.09E+04	1.55E+04	8.37E+03
F17	min	4.78E+03	7.49E+03	1.18E+04	5.73E+03	7.76E+03	6.02E+03	1.57E+04	5.34E+03
F17	std	7.40E+02	1.39E+03	4.06E+04	1.51E+05	1.24E+04	5.50E+04	9.44E+04	8.08E+02
F17	avg	6.12E+03	8.83E+03	5.99E+04	4.03E+04	1.47E+04	5.29E+04	1.03E+05	7.00E+03
F17	median	6.20E+03	8.29E+03	5.64E+04	7.67E+03	1.15E+04	2.73E+04	6.87E+04	6.88E+03
F18	min	9.60E+05	2.06E+06	6.94E+06	5.71E+05	5.18E+06	1.49E+06	1.14E+07	2.87E+06
F18	std	1.13E+06	2.27E+06	1.73E+07	4.94E+07	4.87E+06	4.87E+06	1.98E+07	4.01E+06
F18	avg	3.02E+06	6.21E+06	2.88E+07	1.46E+07	1.30E+07	6.75E+06	3.74E+07	7.08E+06
F18	median	2.91E+06	5.84E+06	2.51E+07	1.80E+06	1.26E+07	6.06E+06	3.53E+07	5.64E+06
F19	min	2.75E+03	2.51E+06	3.07E+09	8.42E+06	2.87E+07	7.19E+07	4.43E+09	4.33E+06
F19	std	1.16E+05	9.74E+06	2.91E+09	5.26E+09	5.34E+07	3.70E+09	2.23E+09	8.66E+06
F19	avg	5.42E+04	1.15E+07	9.05E+09	2.49E+09	1.15E+08	4.01E+09	8.86E+09	1.70E+07
F19	median	1.32E+04	9.15E+06	9.08E+09	1.47E+08	1.12E+08	2.57E+09	8.96E+09	1.55E+07
F20	min	4.28E+03	5.15E+03	5.98E+03	3.99E+03	5.09E+03	4.33E+03	6.03E+03	4.72E+03
F20	std	5.49E+02	6.41E+02	5.05E+02	8.18E+02	7.07E+02	5.41E+02	5.40E+02	5.96E+02
F20	avg	5.46E+03	6.80E+03	7.31E+03	5.64E+03	6.90E+03	5.50E+03	7.11E+03	5.93E+03
F20	median	5.50E+03	6.89E+03	7.36E+03	5.60E+03	6.95E+03	5.50E+03	7.20E+03	5.97E+03
F21	min	1.02E+04	1.55E+04	1.70E+04	1.07E+04	1.49E+04	1.34E+04	1.71E+04	1.30E+04
F21	std	1.14E+03	9.11E+02	6.12E+02	2.05E+03	1.41E+03	9.72E+02	7.67E+02	7.90E+02
F21	avg	1.25E+04	1.74E+04	1.85E+04	1.46E+04	1.74E+04	1.52E+04	1.83E+04	1.50E+04
F21	median	1.25E+04	1.74E+04	1.85E+04	1.44E+04	1.73E+04	1.54E+04	1.83E+04	1.50E+04
F22	min	4.40E+04	6.30E+04	6.95E+04	4.71E+04	6.74E+04	4.86E+04	6.61E+04	5.60E+04
F22	std	9.67E+03	5.14E+03	4.81E+03	5.59E+03	3.88E+03	3.33E+03	5.43E+03	4.28E+03
F22	avg	5.76E+04	7.13E+04	8.17E+04	5.40E+04	7.52E+04	5.57E+04	8.13E+04	6.69E+04
F22	median	5.41E+04	7.02E+04	8.34E+04	5.29E+04	7.58E+04	5.68E+04	8.33E+04	6.70E+04
F23	min	1.19E+04	1.97E+04	1.98E+04	1.68E+04	1.88E+04	1.74E+04	1.95E+04	1.69E+04
F23	std	1.81E+03	2.67E+03	1.09E+03	2.80E+03	2.08E+03	1.15E+03	1.16E+03	1.95E+03
F23	avg	1.46E+04	2.51E+04	2.14E+04	1.97E+04	2.33E+04	1.96E+04	2.14E+04	2.10E+04
F23	median	1.47E+04	2.55E+04	2.13E+04	1.90E+04	2.29E+04	1.93E+04	2.12E+04	2.11E+04
F24	min	1.80E+04	2.03E+04	2.20E+04	1.86E+04	2.07E+04	1.90E+04	2.19E+04	1.87E+04
F24	std	6.34E+02	7.58E+02	4.08E+02	1.40E+03	1.27E+03	7.19E+02	4.07E+02	6.52E+02
F24	avg	1.95E+04	2.21E+04	2.28E+04	2.03E+04	2.28E+04	2.10E+04	2.29E+04	2.02E+04
F24	median	1.95E+04	2.22E+04	2.27E+04	2.01E+04	2.25E+04	2.11E+04	2.30E+04	2.02E+04
F25	min	3.79E+03	4.01E+03	1.69E+04	7.79E+03	6.64E+03	7.58E+03	1.76E+04	4.25E+03
F25	std	1.40E+03	2.15E+02	1.58E+03	6.99E+03	7.28E+02	2.26E+03	1.01E+03	1.43E+02
F25	avg	5.33E+03	4.40E+03	1.93E+04	1.42E+04	8.14E+03	1.25E+04	1.91E+04	4.49E+03
F25	median	4.91E+03	4.41E+03	1.93E+04	1.10E+04	8.05E+03	1.20E+04	1.88E+04	4.49E+03
F26	min	2.18E+04	2.25E+04	3.15E+04	2.67E+04	3.23E+04	2.73E+04	3.10E+04	2.45E+04
F26	std	2.49E+03	6.12E+03	4.17E+03	2.40E+03	3.13E+03	3.19E+03	4.27E+03	1.62E+03
F26	avg	2.73E+04	3.53E+04	3.77E+04	3.29E+04	3.80E+04	3.49E+04	3.86E+04	2.86E+04
F26	median	2.80E+04	3.52E+04	3.71E+04	3.32E+04	3.73E+04	3.49E+04	3.90E+04	2.84E+04
F27	min	3.49E+03	4.42E+03	6.77E+03	4.56E+03	4.51E+03	4.81E+03	6.47E+03	4.42E+03
F27	std	1.58E+03	1.03E+03	9.36E+02	8.36E+02	8.45E+02	6.38E+02	7.85E+02	9.50E+02
F27	avg	4.86E+03	5.58E+03	8.75E+03	5.41E+03	6.01E+03	5.89E+03	8.10E+03	5.42E+03
F27	median	4.41E+03	5.24E+03	8.76E+03	5.26E+03	5.84E+03	5.82E+03	8.15E+03	5.13E+03
F28	min	3.96E+03	4.20E+03	1.68E+04	8.26E+03	8.22E+03	1.16E+04	1.89E+04	4.40E+03
F28	std	1.39E+03	2.65E+02	2.04E+03	7.73E+03	1.27E+03	2.68E+03	2.09E+03	5.46E+02
F28	avg	5.43E+03	4.61E+03	2.21E+04	1.56E+04	1.10E+04	1.71E+04	2.24E+04	5.62E+03
F28	median	5.28E+03	4.53E+03	2.19E+04	1.27E+04	1.08E+04	1.68E+04	2.24E+04	5.61E+03
F29	min	6.98E+03	1.22E+04	2.10E+04	1.04E+04	1.31E+04	1.06E+04	2.94E+04	9.53E+03
F29	std	7.76E+02	1.34E+03	2.75E+04	5.88E+04	3.56E+03	8.70E+03	3.61E+04	8.78E+02
F29	avg	8.06E+03	1.48E+04	5.67E+04	3.35E+04	1.85E+04	1.70E+04	5.83E+04	1.09E+04
F29	median	7.86E+03	1.49E+04	4.74E+04	1.27E+04	1.89E+04	1.52E+04	4.76E+04	1.10E+04
F30	min	1.79E+05	6.57E+07	1.93E+10	8.08E+07	3.86E+08	2.76E+09	1.56E+10	5.52E+07
F30	std	2.37E+08	1.39E+08	3.89E+09	6.08E+09	5.07E+08	4.92E+09	3.40E+09	4.76E+07
F30	avg	7.03E+07	2.52E+08	2.53E+10	3.37E+09	1.41E+09	1.08E+10	2.58E+10	1.45E+08
F30	median	8.84E+05	2.52E+08	2.50E+10	8.65E+08	1.37E+09	1.07E+10	2.66E+10	1.40E+08

**Table 5:** Ranking matrix of 8 algorithms CEC2017 (Dim = 100).

Function	HSHLOA	HLOA	DBO	BKA	WOA	POA	GWO	HHO
F1	3	1	7	5	4	6	8	2
F3	3	5	7	1	8	2	6	4
F4	2	1	7	5	4	6	8	3
F5	1	5	7	2	6	3	8	4
F6	1	5	6	3	8	2	7	4
F7	1	7	4	2	6	3	5	8
F8	1	5	8	2	6	3	7	4
F9	1	4	8	2	7	3	6	5
F10	1	5	7	3	6	2	8	4
F11	2	3	6	1	8	4	7	5
F12	3	2	7	5	4	6	8	1
F13	3	2	8	5	4	6	7	1
F14	1	3	8	4	6	5	7	2
F15	1	2	8	5	4	6	7	3
F16	1	5	8	3	6	4	7	2
F17	1	3	7	5	4	6	8	2
F18	1	2	7	6	5	3	8	4
F19	1	2	8	5	4	6	7	3
F20	1	5	8	3	6	2	7	4
F21	1	5	8	2	6	4	7	3
F22	3	5	8	1	6	2	7	4
F23	1	8	5	3	7	2	6	4
F24	1	5	6	3	7	4	8	2
F25	3	1	8	6	4	5	7	2
F26	1	5	6	3	7	4	8	2
F27	1	4	8	2	6	5	7	3
F28	2	1	7	5	4	6	8	3
F29	1	3	7	6	5	4	8	2
F30	1	3	7	5	4	6	8	2

**Table 6:** Comparison of Wilcoxon rank-sum results for CEC2017 (Dim = 100).

Function	HLOA	DBO	BKA	WOA	POA	GWO	HHO
F1	3.02E−11	3.02E−11	2.15E−10	4.06E−02	3.02E−11	3.02E−11	3.02E−11
F3	2.71E−01	3.34E−11	1.11E−06	3.02E−11	1.01E−08	3.02E−11	1.76E−01
F4	3.11E−01	3.02E−11	3.02E−11	3.02E−11	3.02E−11	3.02E−11	2.51E−02
F5	3.02E−11	3.02E−11	2.05E−03	3.02E−11	2.67E−09	3.02E−11	8.99E−11
F6	3.34E−11	3.02E−11	3.50E−09	3.02E−11	2.15E−10	3.02E−11	3.02E−11
F7	3.34E−11	4.50E−11	2.00E−05	4.50E−11	7.12E−09	5.49E−11	3.02E−11
F8	3.34E−11	3.02E−11	2.01E−04	3.02E−11	6.72E−10	3.02E−11	8.99E−11
F9	3.02E−11	3.02E−11	2.92E−09	3.02E−11	5.07E−10	3.02E−11	3.02E−11
F10	7.39E−11	3.02E−11	1.17E−05	3.02E−11	1.34E−05	3.02E−11	1.55E−09
F11	9.35E−01	3.02E−11	8.29E−06	3.02E−11	2.64E−01	3.02E−11	1.44E−03
F12	2.12E−01	3.02E−11	4.18E−09	1.19E−06	3.34E−11	3.02E−11	4.04E−01
F13	1.43E−08	3.02E−11	5.07E−10	7.12E−09	3.02E−11	3.02E−11	8.48E−09
F14	2.02E−08	3.02E−11	6.57E−02	3.02E−11	3.50E−09	3.02E−11	7.38E−10
F15	3.02E−11	3.02E−11	3.02E−11	3.02E−11	3.02E−11	3.02E−11	3.02E−11
F16	3.69E−11	3.02E−11	3.16E−10	3.02E−11	3.02E−11	3.02E−11	9.26E−09
F17	1.21E−10	3.02E−11	3.09E−06	4.08E−11	3.47E−10	3.02E−11	6.36E−05
F18	9.06E−08	3.02E−11	8.68E−03	3.34E−11	6.36E−05	3.02E−11	2.38E−07
F19	3.02E−11	3.02E−11	3.02E−11	3.02E−11	3.02E−11	3.02E−11	3.02E−11
F20	3.82E−09	6.07E−11	5.30E−01	8.48E−09	8.88E−01	9.92E−11	3.34E−03
F21	3.34E−11	3.02E−11	1.49E−06	4.50E−11	6.72E−10	3.02E−11	1.55E−09
F22	1.29E−06	2.61E−10	2.58E−01	3.08E−08	7.28E−01	4.20E−10	6.36E−05
F23	3.02E−11	3.02E−11	1.21E−10	3.02E−11	3.69E−11	3.02E−11	5.49E−11
F24	4.08E−11	3.02E−11	1.08E−02	3.02E−11	9.26E−09	3.02E−11	6.36E−05
F25	7.96E−03	3.02E−11	9.92E−11	3.50E−09	4.98E−11	3.02E−11	3.64E−02
F26	1.20E−08	3.02E−11	1.17E−09	3.02E−11	3.16E−10	3.34E−11	1.09E−01
F27	1.89E−04	4.18E−09	2.25E−04	7.22E−06	7.22E−06	3.08E−08	2.53E−04
F28	1.56E−02	3.02E−11	4.08E−11	7.39E−11	3.02E−11	3.02E−11	8.24E−02
F29	3.02E−11	3.02E−11	3.02E−11	3.02E−11	3.02E−11	3.02E−11	6.07E−11
F30	2.83E−08	3.02E−11	1.07E−09	8.99E−11	3.02E−11	3.02E−11	1.73E−07

**Table 7:** Comparison of performance of reinforced concrete beams.

Reinforced Concrete Beams	HSHLOA	HLOA	DBO	BKA	WOA	POA	GWO	HHO
Best	1.59E+02	1.59E+02	1.59E+02	1.59E+02	1.59E+02	1.59E+02	1.59E+02	1.59E+02
Worst	1.59E+02	1.83E+02	1.59E+02	1.67E+02	1.81E+02	1.59E+02	1.62E+02	1.59E+02
Std	1.48E−05	8.58E+00	4.85E−02	2.58E+00	7.76E+00	3.00E−14	8.08E−01	2.12E−01
Mean	1.59E+02	1.66E+02	1.59E+02	1.60E+02	1.69E+02	1.59E+02	1.60E+02	1.59E+02
Median	1.59E+02	1.63E+02	1.59E+02	1.59E+02	1.67E+02	1.59E+02	1.59E+02	1.59E+02
Time	1.71E−01	1.06E−01	5.39E−02	6.50E−02	3.06E−02	5.02E−02	5.39E−02	7.89E−02

**Table 8:** Comparison of performance of three-bar truss structures.

	HSHLOA	HLOA	DBO	BKA	WOA	POA	GWO	HHO
Best	2.64E+02	2.64E+02	2.64E+02	2.64E+02	2.64E+02	2.64E+02	2.64E+02	2.64E+02
Worst	2.64E+02	2.70E+02	2.64E+02	2.64E+02	2.68E+02	2.64E+02	2.65E+02	2.64E+02
Std	3.10E−03	1.86E+00	1.74E−01	7.00E−04	1.46E+00	5.99E−14	2.58E−01	5.54E−02
Mean	2.64E+02	2.65E+02	2.64E+02	2.64E+02	2.65E+02	2.64E+02	2.64E+02	2.64E+02
Median	2.64E+02	2.64E+02	2.64E+02	2.64E+02	2.65E+02	2.64E+02	2.64E+02	2.64E+02
Time	3.70E−01	2.04E−01	1.35E−01	1.94E−01	1.03E−01	1.97E−01	1.25E−01	2.62E−01

**Table 9:** Comparison of design performance for pressure vessel design.

Pressure Vessel Design	HSHLOA	HLOA	DBO	BKA	WOA	POA	GWO	HHO
est	6.37E+03	6.48E+03	6.15E+03	6.09E+03	7.81E+03	6.06E+03	6.17E+03	6.13E+03
Worst	7.54E+03	1.10E+04	6.96E+03	7.54E+03	2.13E+04	7.27E+03	6.96E+03	8.13E+03
Std	3.60E+02	1.35E+03	2.43E+02	4.87E+02	4.72E+03	4.04E+02	2.54E+02	5.84E+02
Mean	6.75E+03	7.42E+03	6.42E+03	6.90E+03	1.18E+04	6.33E+03	6.49E+03	6.98E+03
Median	6.77E+03	7.03E+03	6.34E+03	6.94E+03	1.03E+04	6.09E+03	6.45E+03	7.08E+03
Time	3.47E−01	2.03E−01	1.36E−01	1.90E−01	8.96E−02	1.82E−01	1.41E−01	2.40E−01

## Data Availability

The data that support the findings of this study are available from the corresponding author upon request. There are no restrictions on data availability.

## References

[1] Tang Y., Huang K., Tan Z., Fang M., Huang H. (2024). Multi-subswarm cooperative particle swarm optimization algorithm and its application. Inf. Sci..

[2] Guo Q., Yang X., Li K., Li D. (2025). Parameters identification of magnetorheological damper based on particle swarm optimization algorithm. Eng. Appl. Artif. Intell..

[3] Zhao J., Deng C., Yu H., Fei H., Li D. (2024). Path planning of unmanned vehicles based on adaptive particle swarm optimization algorithm. Comput. Commun..

[4] Shang Q., Tan M., Hu R., Huang Y., Qian B., Feng L. (2025). A multi-stage competitive swarm optimization algorithm for solving large-scale multi-objective optimization problems. Expert Syst. Appl..

[5] Abualigah L., Hussein A.M.A., Almomani M.H., Zitar R., Migdady H., Alzahrani A., Alwadain A. (2024). Improved synergistic swarm optimization algorithm to optimize task scheduling problems in cloud computing. Sustain. Comput. Inform. Syst..

[6] Qin S., Zeng H., Sun W., Wu J., Yang J. (2024). Multi-strategy improved particle swarm optimization algorithm and gazelle optimization algorithm and application. Electronics.

[7] Chen B., Cao L., Chen C., Chen Y., Yue Y. (2024). A comprehensive survey on the chicken swarm optimization algorithm and its applications: State-of-the-art and research challenges. Artif. Intell. Rev..

[8] Wang S., Deng Y., Lan N., Cao L., Cheng Z., Xiong M. (2026). Coverage Optimization Strategy for Wireless Sensor Networks Based on Improved Northern Goshawk Optimization Algorithm. Biomimetics.

[9] Fan C., Wang W., Tian J. (2024). Flexible job shop scheduling with stochastic machine breakdowns by an improved tuna swarm optimization algorithm. J. Manuf. Syst..

[10] Yue Y., Cao L., Chen C., Chen Y., Chen B. (2025). Snake Optimization Algorithm Augmented by Adaptive t-Distribution Mixed Mutation and Its Application in Energy Storage System Capacity Optimization. Biomimetics.

[11] Bagherpour R., Bagherpour G., Mohammadi P. (2025). Application of artificial intelligence in tissue engineering. Tissue Eng. Part B Rev..

[12] Cheng Z., Cao L., Qiu Y., Yue Y. (2026). Chaos-Integrated Difference-Enhanced Greater Cane Rat Algorithm and Its Application. Biomimetics.

[13] Yu Y.F., Wang Z., Chen X., Feng Q. (2025). Particle swarm optimization algorithm based on teaming behavior. Knowl.-Based Syst..

[14] Cao L., Yue Y., Chen Y., Chen C., Chen B. (2025). Sailfish optimization algorithm integrated with the osprey optimization algorithm and cauchy mutation and its engineering applications. Symmetry.

[15] Wu N., Jia D., Li Z., He Z. (2024). Trajectory planning of robotic arm based on particle swarm optimization algorithm. Appl. Sci..

[16] Ni J., Miao J., Zheng Y., Cao L., Qiu Y., Yue Y. (2026). Multi-Strategy Improved Red-Billed Blue Magpie Optimization Algorithm and Its Engineering Applications. Biomimetics.

[17] Feng D., Li Y., Liu J., Liu Y. (2024). A particle swarm optimization algorithm based on modified crowding distance for multimodal multi-objective problems. Appl. Soft Comput..

[18] Wang X., Cao L., Zhang Z., Yue Y., Zhang T., Wang Z. (2025). Coverage optimization strategy for 3D wireless sensor network based on adaptive inertia weight Arctic Puffin Optimization Algorithm. J. Comput. Des. Eng..

[19] Wang L., Hong L., Fu H., Cai Z., Zhong Y., Wang L. (2025). Adaptive distance-based multi-objective particle swarm optimization algorithm with simple position update. Swarm Evol. Comput..

[20] Jiao C., Yu K., Zhou Q. (2024). An opposition-based learning adaptive chaotic particle swarm optimization algorithm. J. Bionic Eng..

[21] Cao L., Li M., Chen K., Yue Y., Qiu Y., Cheng Z. (2026). Multi-Strategy Enhanced White Shark Optimizer for Solving Job Shop Scheduling Problem. Biomimetics.

[22] Gao W. (2025). Application of improved particle swarm optimization algorithm combined with genetic algorithm in shear wall design. Syst. Soft Comput..

[23] Wang H., Cai T., Pedrycz W. (2025). Kriging surrogate model-based constraint multiobjective particle swarm optimization algorithm. IEEE Trans. Cybern..

[24] Luo J., Tang P., Xu P., Zhu S., Zhai W. (2025). Efficient predictive model for high-frequency fatigue life of high-speed railway fastening clips using particle swarm optimization algorithm. Mech. Syst. Signal Process..

[25] Peraza-Vázquez H., Peña-Delgado A., Merino-Treviño M., Morales-Cepeda A., Sinha N. (2024). A novel metaheuristic inspired by horned lizard defense tactics. Artif. Intell. Rev..

[26] Li Y., Zhang J., Jin Z., Qiao W. (2025). A multi-strategy improved horned lizard optimization algorithm and its application in engineering optimization. Int. J. Comput. Intell. Syst..

[27] Kanouni B., Laib A., Krama A., Necaibia S., Guerrero J. (2025). Horned Lizard defense tactics optimization algorithm for precise identification of PEMFC parameters. Fuel Cells.

[28] Yin B., Lu H., Dai L., Ding H. (2026). Multi-Strategy-Enhanced Improved Horned Lizard Optimization Algorithm for Path Planning in Mobile Robots. Algorithms.

[29] Alfawaz O., Mostafa R.R., Khedr A.M. (2025). Localization in Wireless Sensor Networks Based Enhanced Horned Lizard Optimization Algorithm. 2025 IEEE 22nd International Multi-Conference on Systems, Signals & Devices (SSD).

[30] Song H.M., Zhang S.W., Wang J.S., Xing C., Sun Y., Wang Y., Sui X. (2025). Task scheduling of cloud computing system by frilled lizard optimization with time varying expansion mixed function oscillation and horned lizard camouflage strategy. J. Netw. Comput. Appl..

[31] Geng Y., Du Q., Ma W. (2025). An Improved Horned Lizard Optimization Algorithm. 2025 IEEE 6th International Seminar on Artificial Intelligence, Networking and Information Technology (AINIT).

[32] Rezk H., Bouaouda A., Hashim F.A. (2025). A novel improved horned lizard optimization algorithm to identify optimal parameters of adaptive fuzzy logic MPPT for performance boosting of PEM fuel cell. Intell. Syst. Appl..

[33] Abdel-Salam M., Tarek Z., Zhong R., Hu G., Bacanin N. (2026). A Novel Dynamic Horned Lizard Algorithm with Advanced Strategies for High-Dimensional Optimization and Pathology Lung Cancer Image Segmentation. Knowl.-Based Syst..

[34] Gámez M.G.M., Vázquez H.P., Cortez J.A.S., Delgado A. (2026). Tuning a Proportional-Integral-Derivative (PID) controller by the HLOA Algorithm for the Sloshing Dynamics Problem. J. Phys. Conf. Ser..

[35] Pandeeswari M.R.M.A., Rajakumar G. (2025). Deep intelligent technique for person Re-identification system in surveillance images. Pattern Recognit..

[36] Wang M., Song X., Liu S., Zhao X., Zhou N. (2025). A novel 2D Log-Logistic–Sine chaotic map for image encryption. Nonlinear Dyn..

[37] Liu J., Deng Y., Liu Y., Chen L., Hu Z., Wei P., Li Z. (2024). A logistic-tent chaotic mapping Levenberg Marquardt algorithm for improving positioning accuracy of grinding robot. Sci. Rep..

[38] Xie L., Wang Y., Tang S., Huang C., Li Y., Dong K., Song T. (2024). A novel adaptive parameter strategy differential evolution algorithm and its application in midcourse guidance maneuver decision-making. Complex Intell. Syst..

[39] Liu Q., Zhang C., Li Z., Peng T., Zhang Z., Du D., Nazir M. (2024). Multi-strategy adaptive guidance differential evolution algorithm using fitness-distance balance and opposition-based learning for constrained global optimization of photovoltaic cells and modules. Appl. Energy.

[40] Wang J., Liu Y., Rao S., Zhou X., Hu J. (2023). A novel self-adaptive multi-strategy artificial bee colony algorithm for coverage optimization in wireless sensor networks. Ad Hoc Netw..

[41] Liao Y.J., Tarng W., Wang T.L. (2025). The effects of an augmented reality lens imaging learning system on students’ science achievement, learning motivation, and inquiry skills in physics inquiry activities. Educ. Inf. Technol..

[42] Ai C., He S., Fan X. (2023). Parameter estimation of fractional-order chaotic power system based on lens imaging learning strategy state transition algorithm. IEEE Access.

[43] Li W., Luo H., Wang L. (2023). Multifactorial brain storm optimization algorithm based on direct search transfer mechanism and concave lens imaging learning strategy. J. Supercomput..

[44] Xue J., Shen B. (2023). Dung beetle optimizer: A new meta-heuristic algorithm for global optimization. J. Supercomput..

[45] Wang J., Wang W., Hu X., Qiu L., Zang H. (2024). Black-winged kite algorithm: A nature-inspired meta-heuristic for solving benchmark functions and engineering problems. Artif. Intell. Rev..

[46] Nadimi-Shahraki M.H., Zamani H., Asghari Varzaneh Z., Mirjalili S. (2023). A systematic review of the whale optimization algorithm: Theoretical foundation, improvements, and hybridizations. Arch. Comput. Methods Eng..

[47] Trojovský P., Dehghani M. (2022). Pelican optimization algorithm: A novel nature-inspired algorithm for engineering applications. Sensors.

[48] Dada E., Joseph S., Oyewola D., Fadele A., Chiroma H., Abdulhamid S. (2022). Application of grey wolf optimization algorithm: Recent trends, issues, and possible horizons. Gazi Univ. J. Sci..

[49] Heidari A.A., Mirjalili S., Faris H., Aljarah I., Mafarja M., Chen H. (2019). Harris hawks optimization: Algorithm and applications. Future Gener. Comput. Syst..

[50] Agushaka J.O., Akinola O., Ezugwu A.E., Oyelade O.N., Saha A. (2022). Advanced dwarf mongoose optimization for solving CEC 2011 and CEC 2017 benchmark problems. PLoS ONE.

[51] Salgotra R., Sharma P., Kundu K., Raju S., Gandomi A. (2025). Enhancing differential evolution algorithm for CEC 2014, CEC 2017, CEC 2021, and CEC 2022 test suites. Neural Comput. Appl..

